# Modulated control of DNA supercoiling balance by the DNA-wrapping domain of bacterial gyrase

**DOI:** 10.1093/nar/gkz1230

**Published:** 2020-01-17

**Authors:** Matthew J Hobson, Zev Bryant, James M Berger

**Affiliations:** 1 Department of Biophysics and Biophysical Chemistry, Johns Hopkins University School of Medicine, Baltimore, MD 21205, USA; 2 Department of Bioengineering, Stanford University, Stanford, CA 94305, USA; 3 Department of Structural Biology, Stanford University School of Medicine, Stanford, CA 94305, USA

## Abstract

Negative supercoiling by DNA gyrase is essential for maintaining chromosomal compaction, transcriptional programming, and genetic integrity in bacteria. Questions remain as to how gyrases from different species have evolved profound differences in their kinetics, efficiency, and extent of negative supercoiling. To explore this issue, we analyzed homology-directed mutations in the C-terminal, DNA-wrapping domain of the GyrA subunit of *Escherichia coli* gyrase (the ‘CTD’). The addition or removal of select, conserved basic residues markedly impacts both nucleotide-dependent DNA wrapping and supercoiling by the enzyme. Weakening CTD–DNA interactions slows supercoiling, impairs DNA-dependent ATP hydrolysis, and limits the extent of DNA supercoiling, while simultaneously enhancing decatenation and supercoil relaxation. Conversely, strengthening DNA wrapping does not result in a more extensively supercoiled DNA product, but partially uncouples ATP turnover from strand passage, manifesting in futile cycling. Our findings indicate that the catalytic cycle of *E. coli* gyrase operates at high thermodynamic efficiency, and that the stability of DNA wrapping by the CTD provides one limit to DNA supercoil introduction, beyond which strand passage competes with ATP-dependent supercoil relaxation. These results highlight a means by which gyrase can evolve distinct homeostatic supercoiling setpoints in a species-specific manner.

## INTRODUCTION

During DNA replication, transcription and repair, topological challenges in the form of DNA supercoiling or entanglements arise that must be addressed to safeguard genomic integrity (1,2). Topoisomerases are ubiquitous and essential regulators of DNA topology, required both for unlinking newly-replicated sister chromosomes (decatenation) and for maintaining the genome in an appropriately supercoiled state (3,4). DNA supercoiling in particular plays an important role in transcriptional programming and cellular physiology (5); for example, in *Escherichia coli*, the dysregulation of negative supercoiling either genetically or through the use of topoisomerase-targeting drugs impacts the transcription of ∼10% of promoters throughout the genome (6). The type IIA subfamily of topoisomerases both resolves DNA entanglements and regulates DNA supercoiling through a strand passage mechanism whereby one DNA duplex, the transfer (T) segment, is transported through a double-stranded break in a second DNA, the gate (G) segment (4,7). ATP binding and hydrolysis serve to coordinate the opening and closing of three inter-subunit interfaces (termed ‘gates’) that mediate DNA breakage and transport, and to prevent the formation of potentially fatal double strand breaks (8). Although type IIA toposiomerases are capable of binding and hydrolyzing two ATP molecules per cycle, whether ATP-hydrolysis is synchronous, and whether both molecules are always consumed during each strand passage event, has been an area of debate (8–11).

**Figure 1.**
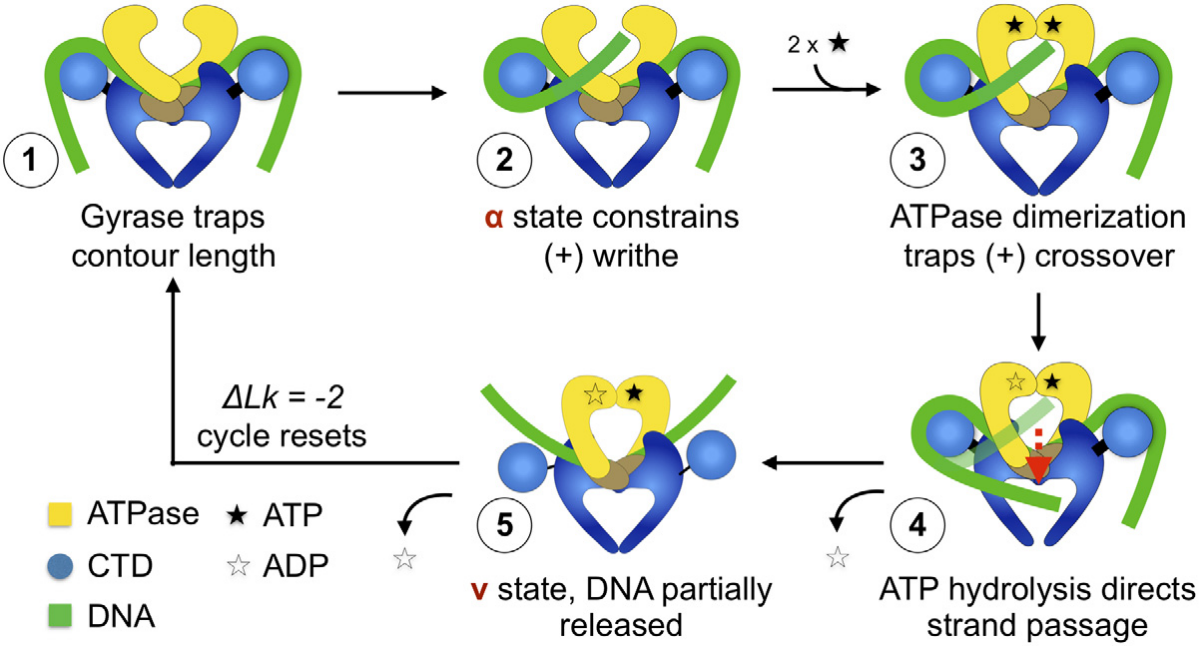
Simplified mechanism of supercoiling by gyrase. DNA supercoiling by gyrase is facilitated by the formation of a chiral wrap prior to strand passage. 1) The GyrA CTD binds and wraps DNA. 2) The wrapped CTD presents a nascent T-segment *in cis* prior to GyrB dimerization. 3–4) ATP binding promotes GyrB dimerization and triggers strand passage. 4–5) The T-segment DNA is partially released and the enzyme resets. ‘α’ refers to the chirally-wrapped state of DNA by gyrase, ‘ν’ to a DNA-bound but CTD-disengaged state (46).

Most bacteria encode two type IIA topoisomerases, topo IV and gyrase, which are responsible for chromosome decatenation and DNA supercoiling, respectively (12). Both enzymes are heterotetramers, separating the ATPase and G-segment binding and cleavage elements into different subunits (ParE and ParC for topo IV and GyrB and GyrA for gyrase). A specialized C-terminal DNA binding domain appended to ParC and GyrA (the ‘CTD’) helps dictate the specialized functions of topo IV and gyrase (13–16). Gyrase in particular is uniquely capable of using ATP turnover to negatively supercoil DNA (17), a reaction accomplished by the formation of a chiral (+) DNA wrap around the CTD prior to strand passage (13–16,18). This wrap ensures that the product of the gyrase reaction is the incorporation of two negative supercoils (Figure 1). The CTD of topo IV, though a close homolog to the GyrA CTD, is unable to fully wrap DNA and thus cannot supercoil DNA (19,20).

**Figure 2.**
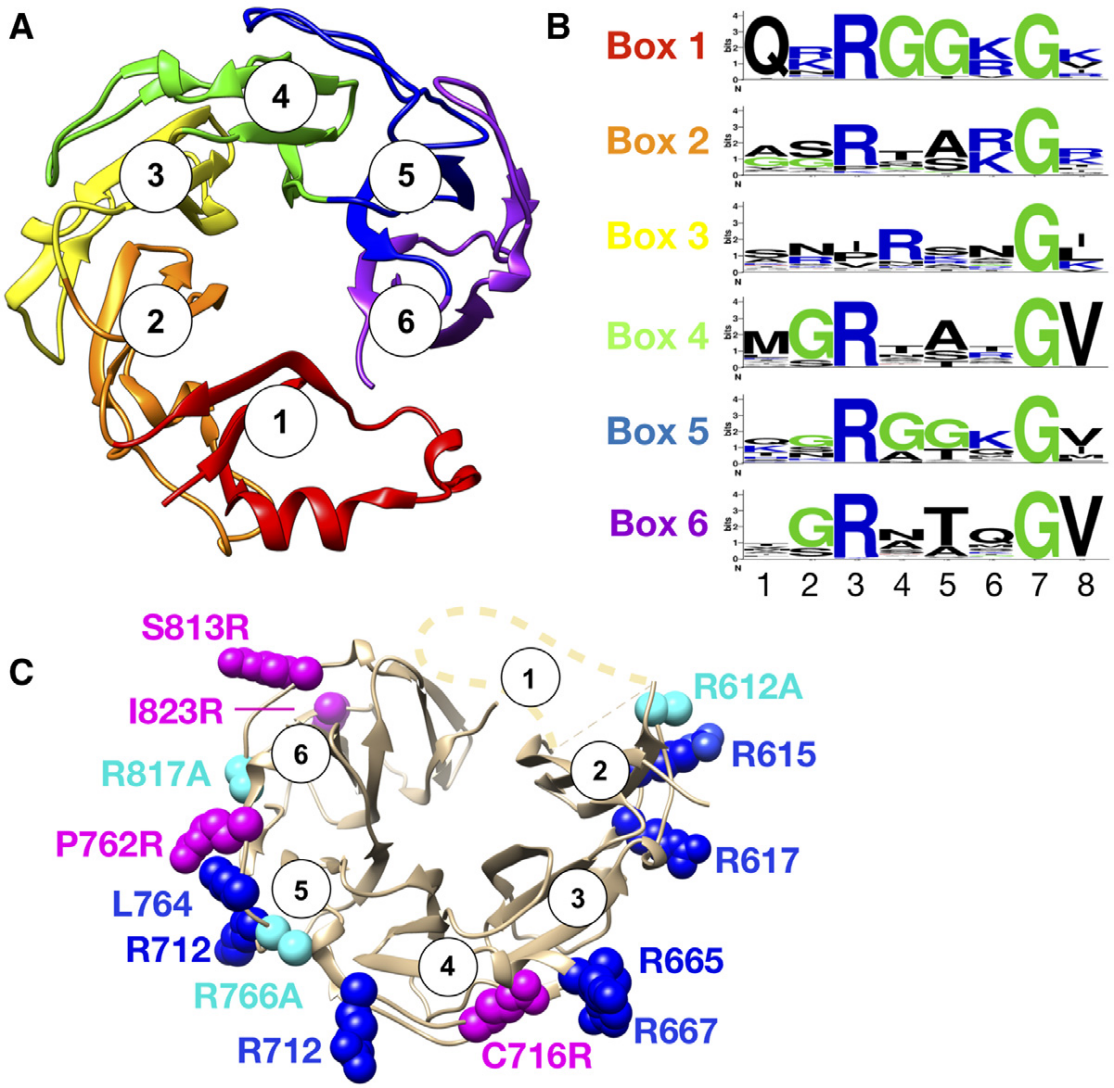
CTD Mutant design and rationale. (**A**) Cartoon representation of the *E. coli* GyrA CTD (PDB: 1ZI0) (14). CTD blades (N- to C-terminal) are numbered 1–6. (**B**) LOGO representation (69) of the GyrA-box repeats within each blade. Profiles are based on an alignment of >1000 GyrA orthologs. The motifs are characterized by high R/K and G content. (**C**) Cartoon of the *E. coli* GyrA CTD highlighting the position of basic residues that are naturally present in the GyrA-box motifs (dark blue spheres). Positions where neutral-to-basic changes were made (C716, P762, S813, I823) are shown in magenta. Positions that where basic charges were removed (R612, R766, R817) are shown in cyan.

Studies have revealed that conserved basic residues which reside at roughly equidistant points along the exterior surface of the ParC CTD help control DNA binding by topo IV and promote the discrimination of different DNA topologies to mediate efficient substrate decatenation (21). DNA binding by the GyrA CTD relies on a similar surface and set of residues to mediate DNA wrapping (16,22). Crystal structures and phylogenetic analyses show that the GyrA CTD consists of a quasi-circular all-β fold composed of six repeating subdomains termed ‘blades’ (Figure 2A) (14,16,19,23). The CTD had been proposed to wrap DNA in a manner analogous to the nucleosome, using basic residues arrayed about its circumference to bend DNA around the fold (16,24,^25^); recent cryo-EM studies have confirmed this hypothesis (22). A conserved (R/K)xxxG amino acid sequence motif on the outer surface of Blade 1, termed the ‘GyrA-box’, is essential for DNA wrapping and supercoiling (26,27). Due to the repetitive nature of the CTD, homologous GyrA-box sequence motifs are present in the other CTD blades as well (28), though are less conserved (Figure 2B).

The fundamental biochemical properties of gyrase, including the rate and steady-state extent of DNA supercoiling (termed the superhelical ‘setpoint’), can vary widely among prokaryotic species. For instance, *E. coli* gyrase can efficiently introduce 1–2 negative supercoils per second into positively supercoiled or relaxed DNA substrates in a highly efficient manner, consuming ∼1–2 molecules of ATP per round of strand passage *in vitro* (29,30). By contrast, purified *Mycobacterium tuberculosis* (Mtb) gyrase is substantially slower (20–50-fold) (31). Mtb gyrase also has a superhelical setpoint that is ∼30% lower than that of *E. coli* (23). DNA wrapping by *E. coli* gyrase is tightly coupled to nucleotide state (32), but less so in gyrases from species such as *M. tuberculosis* or *B. subtilis* (23,33). The coupling between nucleotide binding and release with DNA wrapping has been suggested as a possible explanation for the high efficiency of *E. coli* gyrase's supercoiling reaction (29,30); however, *B. subtilis* gyrase is also fairly efficient at harnessing the energy of ATP to drive the negative supercoiling of DNA, yet does not couple DNA release to nucleotide binding (33). Thus, coordination between DNA wrapping and ATPase status may not be solely responsible for the high efficiency of *E. coli* gyrase.

Several mechanisms have been proposed to explain how the superhelical setpoint of gyrase is defined biophysically. Nöllmann *et al.* have suggested that the maximal extent of DNA supercoiling by gyrase arises from a kinetic competition between ATP-dependent negative supercoiling and ATP-independent supercoil relaxation, and that steady-state supercoiling levels are achieved when the probability of wrapping and passing a T-segment in *cis* equals that of engaging and passing a T-segment in *trans* (34). An alternative hypothesis by Kampranis *et al.* noted that DNA wrap formation occurs with high probability, regardless of the substrate's superhelical density, and proposed that supercoiling is limited by the likelihood of transporting a proximal T-segment across the DNA gate—a probability dictated by the superhelical density and the ability of the enzyme to use ATP binding and hydrolysis to drive the strand passage reaction forward (35). The extent to which gyrase employs one mechanism *versus* the other (or both) remains to be definitively established. A complicating factor is that the GyrA CTD, like that of ParC (21), may act as a DNA topology ‘rheostat’, an element that helps set the maximal extent of DNA supercoiling through an evolutionarily-tunable capacity to stably engage and wrap DNA segments in a supercoiled context (4). From this perspective, the stability of DNA wrap formation may constitute a significant driver of superhelical setpoint, and thus a differentiating factor between the disparate activities of gyrase homologs. Such a concept also has not been formally tested.

To more precisely define the molecular determinants that contribute to the high efficiency and supercoiling extent of gyrase, we set out to directly modulate DNA wrapping by the enzyme by adding or removing probable positively-charged interactions between the *E. coli* GyrA CTD and its DNA substrate, using sequence homology as a guide for selecting targeted amino acids. Biochemical analyses show that these mutants can directly modulate the DNA wrapping propensity, supercoiling rate, supercoiling setpoint, and coupling efficiency of *E. coli* gyrase. Impairment of DNA wrapping is shown to decrease supercoiling and ATPase rate, enzymatic efficiency, and supercoiling extent. By comparison, mutations that improve wrapping lead to slower DNA supercoiling and a decrease in ATPase efficiency, but do not alter gyrase's intrinsic supercoiling setpoint. Interestingly, a mutant gyrase with weakened DNA wrapping shows enhanced decatenation and ATP-dependent negative supercoil relaxation activities compared to the wild type enzyme. Collectively, our findings support the idea that *E. coli* gyrase has evolved to operate at high thermodynamic efficiency (36), balancing the energetics of DNA wrapping/unwrapping with that of nucleotide binding/hydrolysis to drive DNA supercoiling and avoid off-pathway intermediates. Weakening DNA wrapping allows the enzyme to take on more topo IV-like properties, whereas stronger wrapping impairs supercoiling rates and disrupts tight ATPase coupling. These results help account for how DNA wrapping and ATPase efficiency can govern fundamental properties of gyrase that are critical for maintaining supercoiling homeostasis across bacterial species.

## MATERIALS AND METHODS

### Design of CTD mutants

The amino acid sequences of 1100 GyrA CTD and CTD orthologs were obtained from a reference protein database by PSI-BLAST (37) (using *E. coli* GyrA as a query sequence) and aligned using MAFFT (38) (Supplemental Materials). Putative sites for neutral to basic (Φ→R) mutations were identified using a multiple sequence alignment and a >30% sequence conservation cutoff. Only residues on the exterior face of the CTD were considered. Basic residues within each GyrA Box motif were screened using alanine scanning for weaker wrapping activity. Testing of putative weaker wrapping mutants involved assessing the specific activity of each variant (see DNA supercoiling assays in Materials and Methods).

### Cloning, protein expression, and purification

Site directed mutagenesis of the GyrA CTD was conducted using the round the horn protocol (39), using the *E. coli* wild type CTD within the 1C vector (QB3 MacroLab at UC Berkeley) as a template. Full-length GyrA mutants were engineered using round the horn with wild type GyrA in the Pet28b vector. All mutants were confirmed by sequencing.

The wild type and mutant GyrA CTDs constructs were transformed into BL21(DE3)RIL cells, grown in 2xTY media at 37°C and expressed at OD 0.8 for 4 h with 0.25 mM IPTG. GyrB, GyrA and GyrA mutants were expressed in the BL21(DE3)RIL strain. Colonies were selected, grown overnight in MDG media at 37°C and then diluted 1:100 into M9ZB media at 37°C. Once the cells reached OD 1.4, 0.25 mM IPTG was added and protein was expressed at 18°C overnight. For both the GyrA CTDs and full length GyrA constructs, cells were harvested via centrifugation and resuspended in buffer containing 1 M NaCl, 25 mM Tris pH 7.9, 30 mM Imidazole pH 8.0 and 10% glycerol along with protease inhibitors (1 mM PMSF, 1 ug/ml Pepstatin A and 1 ug/ml Leupeptin) [Buffer A1000]. Re-suspended cells were frozen dropwise in liquid nitrogen and kept at −80°C until use.

When purifying all constructs, 0.1 mg/ml of lysozyme and DNAse I were added to thawed cell pellets and cells were lysed using a LM20 microfluidizer or by sonication in the case of small scale purifications (<2 l of cell culture). Following lysis, insoluble material was spun down at 15 000 RPM and discarded. For the MBP–CTD constructs, the soluble lysate fraction was passed over a 5 ml Ni-HiTrap column (GE) and washed with A1000 buffer (2× the volume of lysate per wash). Protein was eluted with buffer containing 150 mM NaCl, 25 mM Tris pH 7.9, 500 mM Imidazole pH 8.0 and 10% glycerol (E150 buffer) and fractions containing protein were concentrated using an Amicon 30 kDa MWCO centrifugal filter (Millipore). Further sample cleanup was conducted using a Sephacryl 5/150 S200 size exclusion column on an AKTA FPLC (GE) and fractions containing protein were verified for purity using SDS-PAGE for analysis. Sizing buffer contained 500 mM KCl, 25 mM Tris pH 7.9 and 10% glycerol (S500). Glycerol concentration was adjusted to 30% to act as a cryo-protectant prior to freezing in liquid nitrogen.

To purify full length GyrB and GyrA constructs, cells were thawed and lysed as with the CTD constructs followed by passage over a nickel column. After washing with A1000 buffer, the column was equilibrated with an additional 10 column volumes of buffer containing 150 mM NaCl, 25 mM Tris pH 7.9 and 10% glycerol (A150). A 5 ml Q-HiTrap column in buffer A150 was then attached and bound protein was eluted off of nickel to Q using E150 buffer. The Q column was then washed with another 10-volumes of A150 buffer and bound protein was eluted off using A1000. Sample was then concentrated and the His-tag was removed via digestion overnight at 4°C using TEV protease (Macro Lab). Following TEV-digestion, the cleaved tag and protease were removed by re-passing over nickel in A1000 buffer and eluent was concentrated. The final purification step was gel filtration over Sephacryl 5/150 S300 size exclusion column. For GyrA, protein-containing fractions were collected, concentrated and frozen similar to the CTD constructs. GyrB was purified identically except for one key detail. GyrB migrates as a monomer and dimer on S300. Importantly, only the ‘light’ monomer fraction was collected as the ‘heavy’ dimer fractions are often contaminated with endogenously expressed GyrA. Following concentration and freezing, GyrB samples were always tested for supercoiling activity indicative of GyrA contamination. If supercoiling activity was detected, the GyrB prep was re-passed over the S300 to remove any contaminating GyrA.

### Topology footprinting assays

DNA wrapping was measured using topology footprinting assays standard to the field. Measurements were conducted in triplicate to ensure reproducibility; representative figures from these replicates are shown here. For measurement of DNA wrapping by the MBP-GyrA CTD constructs, CTDs covering a concentration range of 1.92 uM to 15 nM were co-incubated with 6 nM nicked pSG483 at 37°C for 10 min in a buffer containing either 80 mM potassium glutamate, 50 mM Tris 7.9, 10 mM MgCl_2_, 10 mM DTT, 0.1 mg/ml BSA, 12% glycerol and 2 mM ATP or 300 mM potassium glutamate, 50 mM Tris 7.9, 10 mM MgCl_2_, 10 mM DTT, 0.1 mg/ml BSA, 12% glycerol and 2 mM ATP. 400 units of T4 ligase (NEB) were then added and the reaction mixture was incubated for an additional 60 min to trap any accumulated writhe upon CTD binding. Ligation reactions were stopped with the addition of 1% SDS and 10 mM EDTA and heat inactivation at 65°C for 10 min. Topoisomers were separated using gel electrophoresis—1.4% agarose in 1× TAE buffer (40 mM sodium acetate, 50 mM Tris 7.9, 1 mM EDTA) at 1.7 V/cm for 18 h.

For topology footprinting of full length GyrA and gyrase holoenzyme, *E. coli* ligase was used rather than T4 ligase as the presence of ATP would result in supercoiling of pSG483 following ligation. GyrA alone or gyrase tetramer over a range of 960–30 nM was incubated with 6nM nicked pSG483 at 37°C for 10 min in a buffer containing either 80 mM potassium glutamate, 30 mM Tris 7.9, 4 mM MgCl_2_, 1 mM DTT, 0.05 mg/ml BSA, 12% glycerol and 26 uM NAD or 300 mM potassium glutamate, 30 mM Tris 7.9, 4 mM MgCl_2_, 1 mM DTT, 0.05 mg/ml BSA, 12% glycerol and 26 uM NAD. For the holoenzyme + AMPPNP condition, 2 mM AMPPNP was added following the 10 min co-incubation of gyrase with nicked plasmid. Writhe was trapped upon the addition of 20 U of *E. coli* ligase (NEB) and incubation for 1 h at 37°C. Ligation reactions were stopped with the addition of 1% SDS and 10 mM EDTA and heat inactivation at 65°C for 10 min. Topoisomers were separated using gel electrophoresis—1.4% agarose in 1× TAE buffer at 1.7 V/cm for 18 h.

### DNA supercoiling assays

Supercoiling activity was assessed two ways: (i) the amount of enzyme required to supercoil ∼50% of 7.9 nM pSG483 within 30 min and (ii) the time required to supercoil ∼50% of relaxed plasmid at 1:1 stoichiometry enzyme–plasmid. Measurements were conducted in triplicate to ensure reproducibility; representative figures from these replicates are shown here. For the titration based assays, a titration series of gyrase holoenzyme was added to 7.9 nM pSG483 in a buffer containing 80 mM potassium glutamate, 5.5 mM MgCl_2_, 0.1 mg/ml BSA, 0.5 mM TCEP, 30 mM Tris–HCl pH 7.9 and 18% glycerol. For high salt conditions, the potassium glutamate concentration was increased to 300 mM. Supercoiling reactions were started by adding 2 mM ATP and preformed at 37°C for 30 min. Supercoiling reactions were stopped with the addition of 1% SDS and 10 mM EDTA and topoisomers were separated using gel electrophoresis—1.4% agarose in 1× TAE buffer at 1.7 V/cm for 18 h. The relative specific activities of each gyrase—the amount of enzyme required to convert ∼50% of the relaxed plasmid substrate to supercoiled product over 30 min—were estimated by visual inspection.

For supercoiling time courses, gyrase tetramer and plasmid were incubated 1:1 on ice at 200 nM in buffer containing 500 mM potassium glutamate, 25 mM Tris–HCl pH 7.9, 2.5 mM MgCl_2_, 20% glycerol to ensure a Boltzmann-like distribution of enzyme bound to plasmid. Complex was slowly diluted to a 7.9 nM final concentration in conditions containing either 80 or 300 mM potassium glutamate, 30 mM Tris–HCl pH 7.9, 12% glycerol, 5.5 mM MgCl_2_, 0.5 mM TCEP and 0.1 mg/ml BSA. The reaction mixture was incubated at 37°C for 2 min and supercoiling was initiated by adding ATP to a final concentration of 2 mM. Time points were collected by sampling the mixture and adding it 10:1 to a 10× stopping buffer containing 10% SDS and 100 mM EDTA. Topoisomers were separated using gel electrophoresis—1.4% agarose in TAE buffer 1× at 1.7 V/cm for 18 h and visualized using ethidium bromide staining. 1D chloroquine gels were run to observe negatively supercoiled species in 1.2% agarose in 1X TBE buffer (100 mM boric acid, 100 mM Tris base, 2 mM EDTA) plus chloroquine (Sigma) at 2.2 V/cm for 18 h. Chloroquine was soaked out of the gels with TBE buffer and topoisomers were visualized using ethidium bromide staining. Relative supercoiling rates for each gyrase construct – the amount of time required to convert ∼50% of the relaxed plasmid substrate to supercoiled product – were estimated by visual inspection.

To determine the setpoint of gyrase, 2D gel electrophoresis was performed on a range of topoisomers to count out the topoisomers generated over the course of a supercoiling reaction. A miniaturized version of pUC57-Kan rather than pSG483 was used to maximize resolution between topoismers on gel (Supplemental Materials). A supercoiling timecourse assays were conducted at 80 and 300 mM KGlu with wild type gyrase as described above and samples were run on gel under both native conditions and in the presence of chloroquine to make sure the setpoint had been reached. The C716R and 4R+ mutants were only measured at 300 mM KGlu given their very slow supercoiling rate in 80 mM KGlu. Timepoints were then pooled and cleaned up using phenol chloroform extraction and concentrated via ethanol precipitation and resuspension in 10 ul of TBE buffer + loading dye. The pooled topoisomers were separated using 2D electrophoresis on a 2% agarose 1× TBE gel. The first dimension contained 3 ug/ml chloroquine and was run at 2.2 V/cm for 20 h. The gel was then equilibrated into 1× TBE buffer containing 20 ug/ml chloroquine, rotated 90 degrees, and run for an additional 20 h at 2.2 V/cm. Chloroquine was soaked out of the gels with TBE buffer and topoisomers were visualized using ethidium bromide staining. The maximal extent of negative supercoiling, the setpoint (σ_f_), was determined by counting the toposiomers and dividing the linking number for the most extreme topoisomer seen (ΔLk) by the baseline linking number of the subsrate (Lk_0_ = size of the plasmid in base pairs/10.5) (40).

### ATPase assays

ATPase measurements were conducted using the established enzyme-coupled PK/LDH ATPase assay (41). In this method, ATP regeneration is coupled to the oxidation of NADH. As NADH is converted to NAD, the corresponding decrease in absorption at 340 nM correlates 1:1 to the corresponding hydrolysis of ATP. ATPase rates were calculated using an NADH standard curve equating 340 nm absorption with NADH concentration. Measurements were conducted using a CLARIOStar Omega plate reader. DNA-stimulated ATPase activity was measured at 37°C using 100 nM gyrase tetramer in the presence of saturating DNA (100 ng/ul) over a range of 0–2 mM ATP. The 75 ul reactions volumes contained either 50 or 300 mM potassium glutamate, 50 mM Tris-pH 7.9, 5 mM MgCl_2_, 0.1 mg/ml BSA, 5 mM βME, 2 mM phosphoenolpyruvate, 0.2 mM freshly-made NADH and 1.5 U/ml pyruvate kinase/lactic dehydrogenase mix (Sigma). Three sample replicates were measured for each condition. ATPase data were fit to a pseudo Michaelis–Menten model (*V* = *k*_cat_*[ATP]/(*K*_M_ + [ATP])) in Mathematica. Error bars in the data (Supplemental Figure S6A and B) are reported as standard deviations and errors for the Michaelis–Menten parameters as the deviation of those parameters from the fitted data.

### Decatenation and negative DNA supercoil relaxation assays

Decatenation assays were performed to assess the amount of enzyme required to decatenate 50% of the kDNA substrate (Inspiralis) within 30 min. A titration series of gyrase holoenzyme (or gyrase mutants) was added to 300 ng kDNA in a buffer containing 80 mM potassium glutamate, 5.5 mM MgCl2, 0.1 mg/ml BSA, 0.5 mM TCEP, 30 mM Tris–HCl pH 7.9 and 18% glycerol. Decatenation reactions were started by adding 2 mM ATP and preformed at 37°C for 30 min. Reactions were stopped with the addition of 1% SDS and 10 mM EDTA and topoisomers were separated using gel electrophoresis—1.4% agarose in 1× TAE buffer at 1.7 V/cm for 18 h. Wild type gyrase and the R766A gyrase mutant were compared in their ability to relax negatively-supercoiled DNA. Reactions were conducted in a time dependent format where enzyme was combined 1:1 with negatively supercoiled pSG483 as described above (DNA supercoiling assays). Final reaction conditions consisted of 80 mM potassium glutamate, 30 mM Tris–HCl pH 7.9, 12% glycerol, 5.5 mM MgCl2, 0.5 mM TCEP and 0.1 mg/ml BSA. Wild type and R766A gyrase were both tested in the absence of ATP to compare their ATP-independent relaxation activity. Additionally, ATP-dependent negative supercoil relaxation was measured for the R766A mutant in the presence of 2 mM ATP. Topoisomers were separated using gel electrophoresis—both 1.4% agarose in 1× TAE buffer at 1.7 V/cm for 18 h and 1.2% agarose in 1× TBE buffer plus 1 ug/ml chloroquine at 2.2 V/cm for 18 h to better resolve the relaxation of negative supercoils early in the reaction.

## RESULTS

### Design, selection and preliminary evaluation of GyrA-CTD mutants

To identify residues in the *E. coli* GyrA-CTD that might contribute to DNA binding and wrapping, we first examined sequence alignments of GyrA CTD orthologs for lysine or arginine residues that appear with high frequency on the exterior surface of the domain. *E. coli* GyrA possesses several basic amino acids, particularly in the (R/K)xxxG GyrA-box sequence motifs present in most of the CTD blades. Residues frequently found to be lysine or arginine in bacterial GyrA CTDs, but are neutral in *E. coli* GyrA were chosen as candidates for mutagenesis to strengthen wrapping. One such position, C716, was substituted with arginine, which is found at this location (the GyrA-box motif of blade 4) in >90% of gyrase orthologs. Because it was unclear whether a single point change would noticeably improve DNA wrapping, we also designed a second mutant (‘4R+’), which contains three additional changes to amino acids that are frequently positively-charged in GyrA (P762R, S813R, and I823R) (Figure 2C).

We next designed a set of GyrA mutants with potentially weaker wrapping activities based on similar sequence-guided principles. Here, arginine residues present in the GyrA box-like sequences of each CTD blade that have a high degree of sequence conservation across gyrase orthologs were individually mutated to alanine. Three constructs—R612A, R766A and R817A—were cloned, expressed, purified, and tested to determine their effect on DNA supercoiling. R612A and R817A show no observable effect on DNA supercoiling activity and so were not considered further (Supplementary Figure S1). By contrast, R766A displayed a lower specific activity and setpoint and thus was chosen for additional study. Interestingly, R766 (blade 5) is the most conserved arginine (>98%) within all GyrA-box motifs outside of the parent element in blade 1 and the GyrA box motif in blade 5 has been shown to be important for DNA supercoiling and decatenation by *M. tuberculosis* gyrase (42).

### Alterations to the GyrA CTD can impact DNA wrapping propensity

We initially set out to determine the effect of our GyrA CTD mutations on DNA wrapping using topology footprinting. In this assay, DNA writhe introduced by the binding of a protein to a nicked plasmid is trapped by ligation, and the resultant topoisomer distribution is visualized using native gel electrophoresis to assess the degree of wrapping. Experiments were first conducted with GyrA-CTD constructs (residues 531–853) to examine how charge insertion/deletion affects DNA wrapping by the isolated domain (Materials and Methods). The C716R mutation has a negligible impact on DNA wrapping relative to wild type, while the more extensive quadruple charge addition mutant (4R+) wraps DNA only moderately better (Supplementary Figure S2A). By comparison, the single charge elimination mutant (R766A) exhibits very little DNA wrapping propensity. Repeating the assay at higher ionic strength (80 versus 300 mM potassium glutamate (KGlu)) to establish how elevated salt concentrations might impact wrapping activity reveals a similar loss of activity for the charge removal mutant, and somewhat more-enhanced wrapping activity for the 4R+ charge addition construct. These findings indicate that the wrapping capacity of the wild type *E. coli* GyrA CTD is near maximum, but that this activity can be markedly weakened by just a single amino acid change.

**Figure 3.**
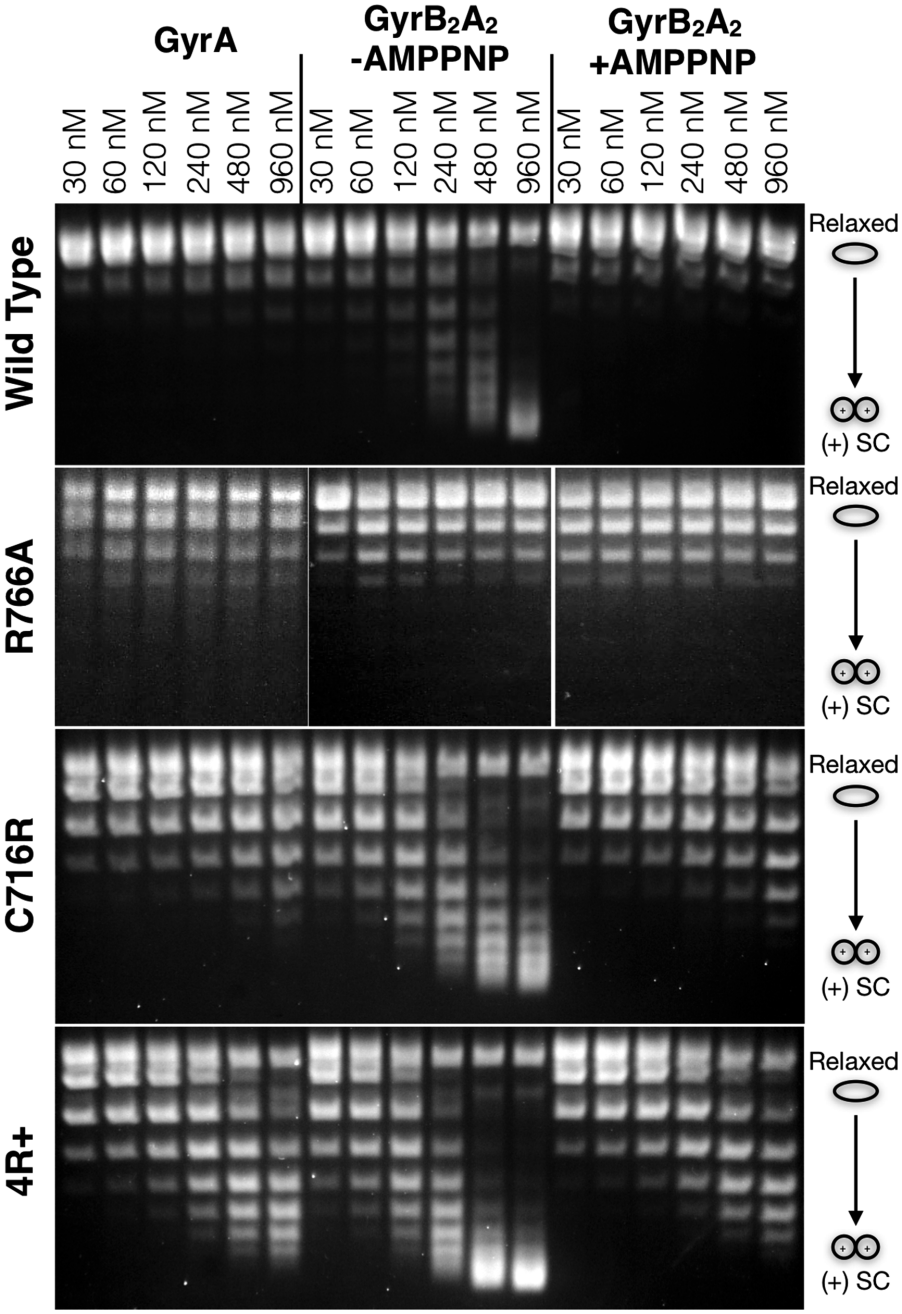
Topology footprinting by GyrA CTD mutants. Native agarose gels showing the DNA wrapping behavior of the purified R766A, C716R and 4R+ mutant GyrA subunits and gyrase holoenzymes compared to the wild type protein. Sequential lanes correspond to increasing protein concentrations.

Topology footprinting experiments were next conducted with our mutant panel in the context of both the full-length GyrA subunit and the gyrase holoenzyme. For reconstituted gyrase, experiments were carried out both with and without the non-hydrolyzable ATP analog AMPPNP (32). *Escherichia coli* GyrA does not observably wrap DNA on its own (13), due to a repressive function located in its acidic C-terminal tail (43); however, when reconstituted with GyrB, the gyrase holoenzyme robustly wraps DNA, but only in the absence of AMPPNP (Figure 3) (32,35,44). Compared to its native counterpart, the R766A mutant shows little ability to wrap DNA, either as a GyrA dimer or in the context of the holoenzyme, regardless of whether nucleotide was present. This finding is consistent with topology footprinting data for the isolated CTD showing that the loss of the arginine side chain at this position in blade 5 effectively ablates the ability of the domain to stably trap DNA writhe (Supplementary Figure S2). By contrast, both the C716R and the 4R+ mutant display more extensive wrapping relative to wild type in the context of GyrA alone or in conjunction with the gyrase holoenzyme and AMPPNP; the degree of wrapping by the quadruple charge mutant further exceeds that of the single charge addition. These observations demonstrate that adding conserved basic residues to the perimeter of the GyrA CTD can increase wrapping propensity in the context of both the full-length subunit and the reconstituted gyrase A_2_B_2_ tetramer. Notably, the C716R and the 4R+ mutants still display weakened wrapping activity in the presence of AMPNP compared to the apo condition. This behavior indicates that the coupling between nucleotide binding and DNA wrapping is diminished from the native state by the charge addition mutations, though not altogether eliminated. DNA binding measurements conducted by electrophoretic mobility shift assay (EMSA) showed that these differences in wrapping are not due to an inability of the GyrA mutants to bind DNA (Supplementary Figure S3, Supplemental Materials).

### The rate of ATP-dependent, negative supercoiling of DNA is sensitive to the wrapping propensity of the GyrA CTD and is enhanced by high ionic strength

**Figure 4.**
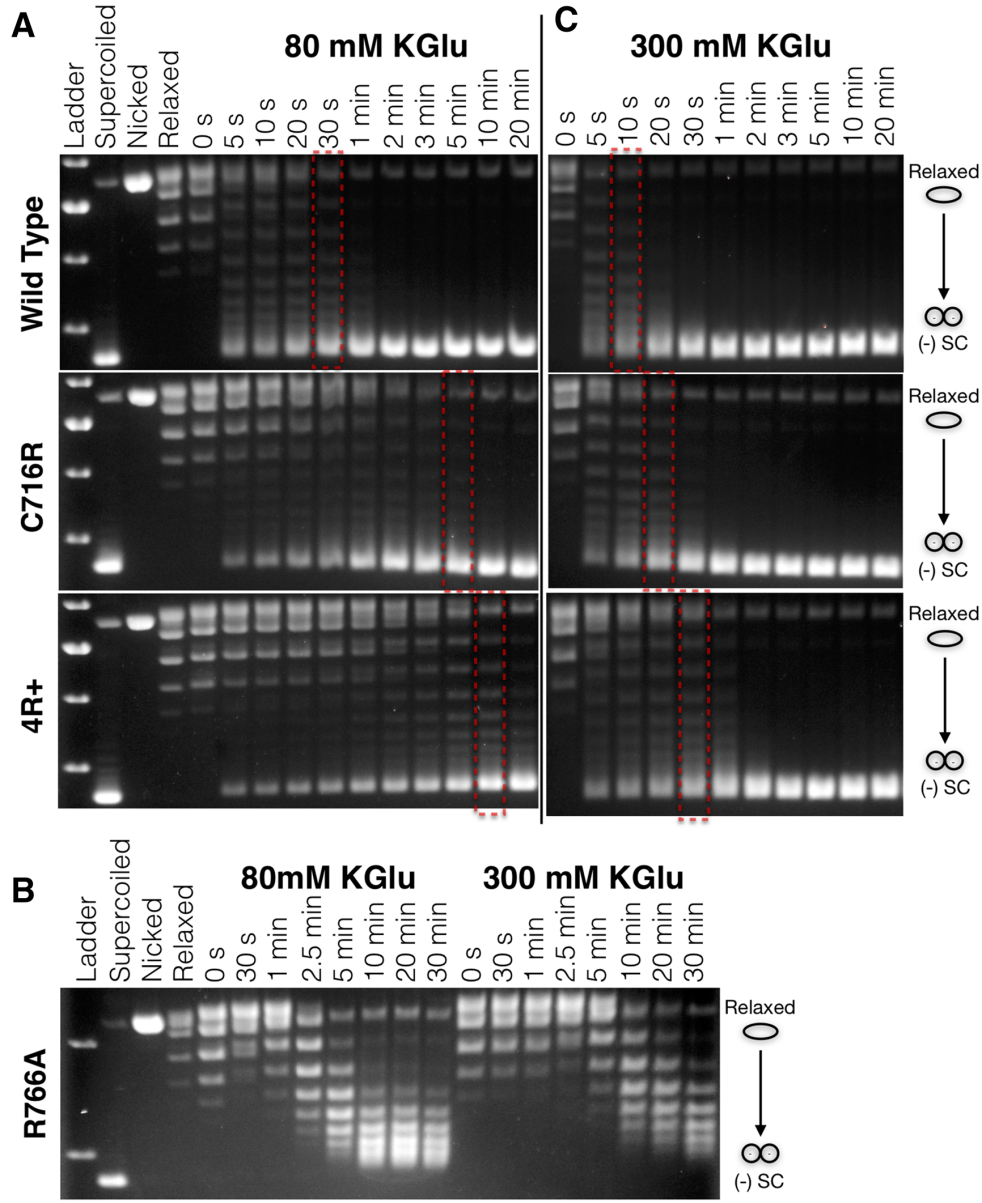
The DNA wrapping propensity of the GyrA CTD impacts DNA supercoiling rate. (**A**) DNA supercoiling timecourse assays for the C716R and 4R+ mutants compared to wildt ype gyrase in the presence of 80 mM KGlu. Dashed boxes denote timepoints with comparable levels of supercoiling. (**B**) Supercoiling timecourses for R766A gyrase at 80 and 300 mM KGlu. (**C**) DNA supercoiling timecourses for the C716R and 4R+ mutants compared to wild type gyrase in the presence of 300 mM KGlu. The molar ratio of enzyme:DNA in all reactions is 1:1.

Having established that our GyrA mutants affect DNA wrapping, we next set out to determine the impact of the charge substitutions on the rate of DNA supercoiling by gyrase using native gel electrophoresis. Wild type gyrase is observed to negatively supercoil >50% of the substrate DNA at a 1:1 enzyme:plasmid ratio in ∼20 s (Figure 4A). This rate corresponds to approximately one strand passage event per second, a value in good agreement with single molecule measurements (45,46). By comparison, the C716R and 4R+ mutants are ∼10- and 20-fold slower, respectively, in catalyzing DNA supercoiling. Negative DNA supercoiling by the R766A mutant is found to be far slower than wild type gyrase (>100-fold) and additionally appears to stall after only a few cycles (Figure 4B). These results demonstrate that both increased and decreased wrapping capacity can markedly influence how quickly gyrase introduces supercoils into DNA.

Given that many protein-DNA interactions are sensitive to electrostatic environment (47), we suspected that the equilibrium between wrapped and unwrapped states of gyrase and our panel of CTD mutants might be salt sensitive and that ionic strength would in turn influence negative supercoiling efficacy. Supercoiling rate measurements were therefore repeated under higher salt conditions (300 mM versus 80 mM potassium glutamate) with the expectation that increasing the ionic strength would weaken the CTD-DNA interactions, potentially relieving the rate defect exhibited by the C716R and 4R+ mutants due to tighter wrapping. Consistent with this prediction, the supercoiling rates of the C716R and 4R+ mutants both increase substantially, reaching near wild type levels (Figure 4C). By contrast, the rate of the R766A mutant decreases even further (∼2-fold) (Figure 4B). Interestingly, wild type gyrase also exhibits a moderate increase in supercoiling rate at the elevated salt concentration (∼3-fold). A repeat of the topology footprinting assays confirms that the C716R and 4R+ mutants wrap DNA at elevated salt concentrations more effectively than wild type protein both in the context of GyrA alone and as a fully reconstituted gyrase in the presence of AMPPNP (Supplementary Figure S4); indeed, the degree of wrapping by the mutants, and even wild type gyrase in the +AMPPNP condition, appears to be slightly increased under this condition compared to low-salt reactions.

### Salt concentration has a more pronounced effect on increasing gyrase supercoiling setpoint than increasing wrapping propensity

**Figure 5.**
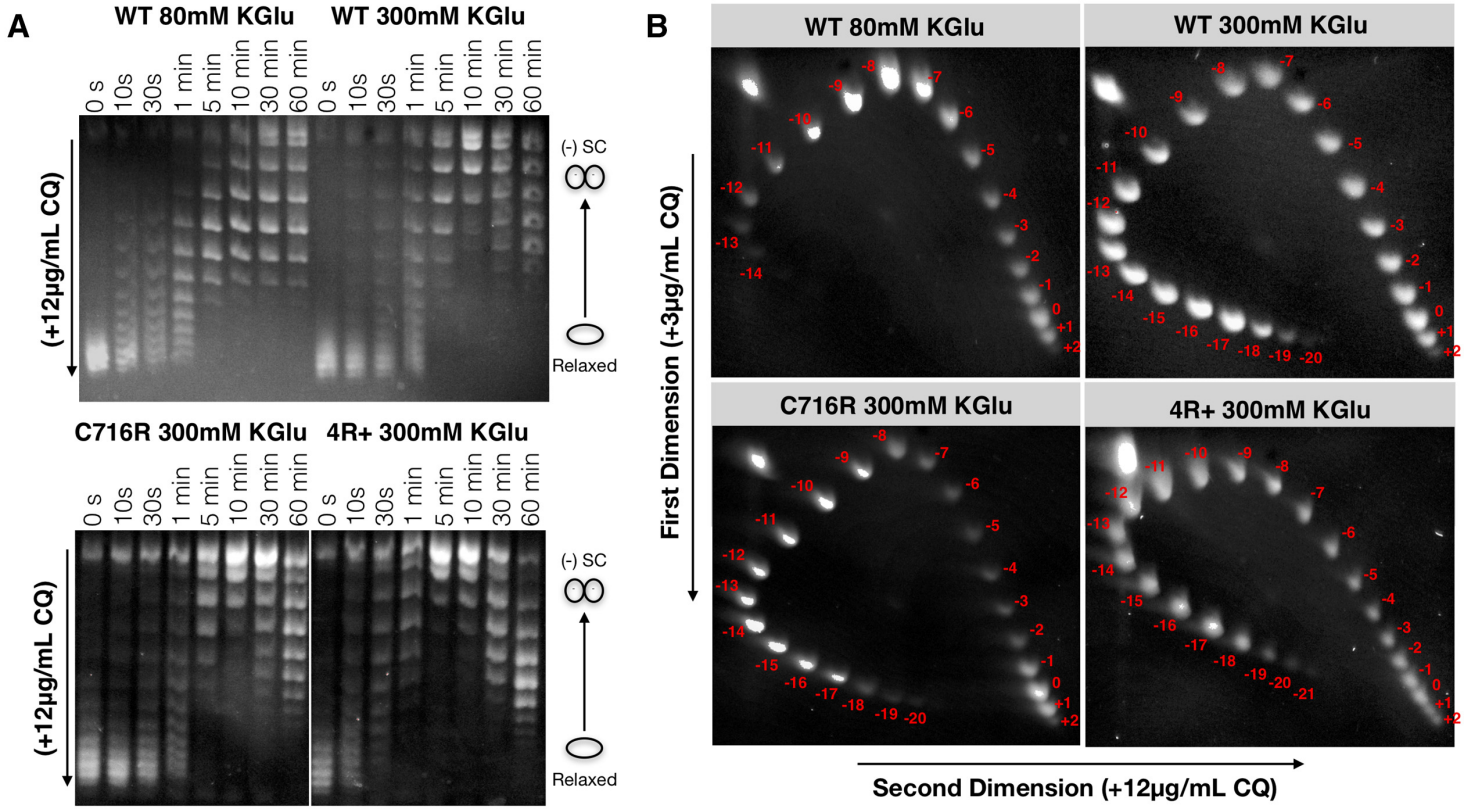
High ionic strength markedly increases the extent of negative supercoiling, whereas increasing wrapping propensity has only a marginal potentiating effect. (**A**) Timecourse reactions showing that DNA supercoiling has reached completion by 30 min. Topoisomer distributions are shifted by the inclusion of 12 μg/ml chloroquine in the gel and running buffer to resolve supercoiled species. The molar ratio of enzyme to DNA is 1:1. (**B**) 2D chloroquine gels quantitate supercoiling setpoint. Gyrase's setpoint is increased by >30% at high KGlu concentrations. Increasing the wrapping propensity of the CTD has a marginal effect on setpoint. Setpoints are calculated using the formula σ_F_ = ΔLk / 161 where 161 is the Lk_0_ for our 1.7 kb plasmid and ΔLk is the excess linking number for the most extreme topoisomer seen. The ΔLk's of −14, −20 and −21 therefore yield σ_F_s of −0.09, −0.12 and −0.13 for WT (80 mM Kglu), WT & C716R (300 mM KGlu) and 4R+ (300 mM KGlu) respectively.

Gyrase orthologs from different bacterial species have been reported to supercoil DNA to different maximal extents (48). Likewise, reaction conditions—i.e., ATP/ADP ratios or the addition of polyamines (49–51)—can also affect the setpoint of gyrase. To determine whether the observed increase in enzymatic efficiency with 300 mM KGlu affects the maximal extent to which gyrase can supercoil DNA, we turned to 2D-gel electrophoresis, a method for quantifying the superhelical density of a plasmid in which an agarose gel is run in two orthogonal directions in the presence of different amounts of an intercalating agent (e.g. chloroquine) (40). Given that the setpoint for gyrase reflects its steady-state maximum for supercoil introduction, it was necessary to ensure that our gyrase activity assays had reached such a point. We therefore first generated topoisomer distributions over different periods of time and visualized the reaction using 1D-chloroquine gels to confirm that they had run to completion (Figure 5A). Analysis of the 2D-gels (Figure 5B) shows that elevated salt concentration (300 mM KGlu) increases the maximal superhelical density (σ_f_) of DNA following gyrase treatment by roughly 30–40% (σ_f_ ≅ −0.12 versus −0.09; see Materials and Methods) compared to lower salt conditions (80 mM KGlu). This result is consistent with a study by Kozyavkin *et al.*, who showed that the addition of various cationic species increases the setpoint of *E. coli* gyrase (51). One point of divergence, however, is that potassium chloride (KCl) was shown to decrease gyrase setpoint as concentrations approached 100 mM, whereas we observed increased activity under even more elevated salt conditions. Reasoning that the difference between our result and that of Kozyavkin *et al.* might be due to the type of anion present in the reactions, we conducted topology footprinting and DNA supercoiling assays at 80 and 300 mM potassium chloride; under these conditions, the higher salt concentration is quite detrimental to both functions (Supplementary Figure S5). These measurements indicate that, as with many DNA-binding proteins and enzymes, gyrase is much more tolerant of glutamate as compared to chloride in supporting activity (52). Glutamate, in addition to being a more physiological salt, appeared less likely to impair DNA binding, which may be attributable to differences in protein–anion interactions (47,52).

To test whether the stimulatory effect of increased KGlu levels on DNA supercoiling was specific to *E. coli* gyrase, we assessed how a different homolog, *Mtb* gyrase, responded to different concentrations of the salt. *Mtb* gyrase has been shown previously to supercoil DNA both more slowly and less extensively than *E. coli* gyrase (23,31). Supercoiling reactions were carried out with the *Mtb* enzyme using either 80 or 300 mM potassium glutamate, and then run on native and 1D chloroquine gels (Supplementary Figure S6). As with its *E. coli* counterpart, *Mtb* gyrase displays a substantial increase (∼50%) in the degree to which it can negatively supercoil DNA at the higher ionic strength. In addition, elevated salt also substantially increases the rate of DNA supercoiling, in a manner reminiscent of the effect of higher KGlu on the 4R+ mutant. The enhancing effect of elevated potassium concentration on gyrase activity is therefore not specific to the *E. coli* enzyme (47).

The mutant gyrase constructs created here allow us to test how the stability of DNA wrapping might help define the maximal extent to which gyrase can underwind DNA. The R766A mutant displays a marked reduction in the degree of DNA supercoiling that it catalyzes compared to wild type gyrase (Figure 4, σ_f_ ≅ −0.03 versus −0.09 at 80 mM KGlu); weakening DNA wrapping thus directly lowers the setpoint of the enzyme. By comparison, strengthening DNA wrapping had a minimal effect on the setpoint. Indeed, the final supercoiled product produced by the C716R and 4R+ gyrase constructs is nearly identical to wild type (Figure 5B) in the presence of 300 mM KGlu (supercoiling with 80 mM KGlu was not evaluated given the slow rate of the C716R and 4R+ mutants). Although both wild type and C716R gyrase are able to introduce roughly 20 negative supercoils into a 1.8 kb plasmid, the 4R+ mutant is capable of undergoing only ∼1–2 additional cycles of supercoiling before stalling. The setpoint of *E. coli* gyrase is therefore not limited by its ability to stably wrap DNA under regimes of high superhelicity, although this value can be decreased if wrapping is severely weakened.

### DNA wrapping propensity impacts the coupling between ATP turnover and strand passage

*Escherichia coli* gyrase exhibits tight coupling between ATP hydrolysis and strand passage, consuming ∼2 molecules of ATP per cycle when bound to a relaxed DNA substrate (11,29,30). As the substrate becomes more underwound, the extent of this coupling is reduced, resulting in futile cycling (i.e. ATP turnover without net negative supercoiling) and a slower supercoiling rate (29,30,35,44,50). Since altering the wrapping propensity of the GyrA CTD can affect both the DNA supercoiling rate and setpoint of the gyrase holoenzyme, we set out to determine whether wrapping exerts a commensurate effect on ATPase function. ATPase activity was measured using a real-time coupled assay at both low and high KGlu concentrations. Two different DNA substrates were used, a nicked plasmid, which cannot build up any superhelical tension, and negatively-supercoiled plasmid obtained from *E. coli* (σ ≅ −0.06), which should resist gyrase action. Basal ATPase rates, conducted in the absence of DNA, were also measured.

**Figure 6.**
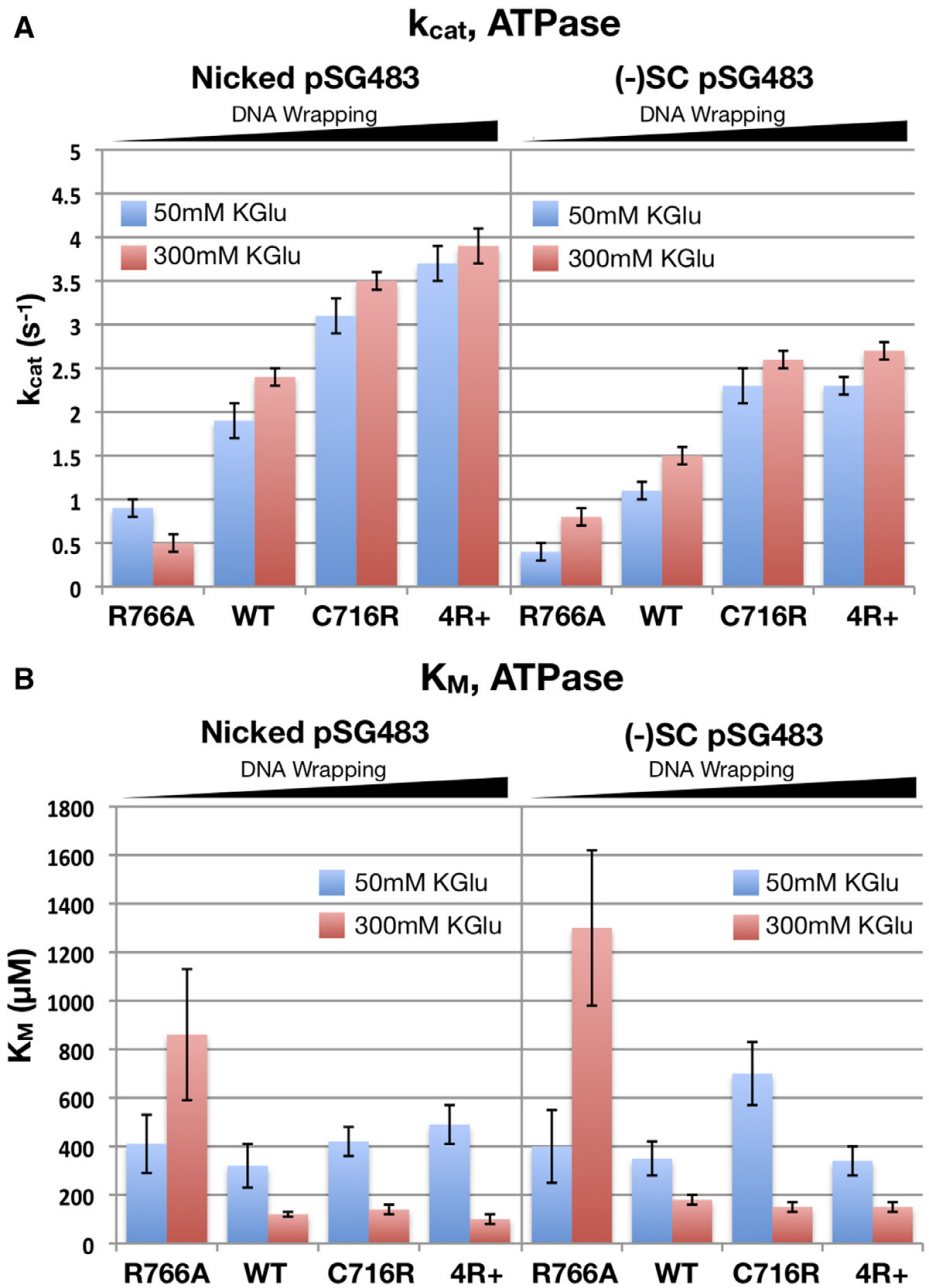
Effect of DNA wrapping and potassium concentration on gyrase ATPase activity. Kinetic parameters derived from Michaelis–Menten fits of ATPase data collected for wild type gyrase and CTD mutants on different DNA substrates at different KGlu concentrations. The molar ratio of enzyme to DNA ‘binding sites’ (300 bp) in all reactions is ∼1:5. The DNA concentrations used in the assays (100 nM) are above the apparent K_D_ of the enzyme (Supplementary Figure S3). (**A**) *k*_cat_ of ATP hydrolysis. *k*_cat_ correlates roughly with DNA wrapping strength and is largely unaffected by high potassium glutamate concentration. Comparatively, ATP-hydrolysis rates are higher on relaxed DNA substrates than on negatively supercoiled DNA (σ = −0.06) (though they are still on the order of typical WT *E. coli* gyrase rates). (**B**) *K*_M_ of ATPase activity. *K*_M_ remains relatively the same regardless of wrapping propensity and the DNA topology of the substrate. High potassium glutamate concentration reduces the *K*_M_ roughly 3-fold relative to low salt, excepting R766A which exhibits DNA-free like behavior (see Supplemental Table S1) at 300 mM KGlu for both DNA substrates.

Similar to previous studies of gyrase (29,53), the observed ATPase rates for all constructs display a pseudo-Michaelis–Menten profile and exhibit a ∼3- to 4-fold increase in rate upon addition of DNA (Supplementary Figure S7). Inspection of the ATPase data revealed several notable, mutant-specific trends (Figure 6). For example, *k*_cat_ increases in a manner that roughly correlates with the wrapping strength of the CTD (R766A < wild type < C716R < 4R+). The topological state of the DNA substrate also affects ATPase rate, with negatively supercoiled DNA lowering *k*_cat_ by ∼30–40% for wild type gyrase and the two charge addition mutants (the weakly-wrapping R766A mutant exhibits *k*_cat_ and *K*_m_ values close to that of the basal ATPase rate, ∼0.5 s^−1^ and >600 uM respectively) (Supplemental Table S1). Collectively, these trends are consistent with the idea that ATPase rate is influenced at least in part by the frequency in which a T-segment is captured by the ATPase domains (7,30) (with the C716R and 4R+ mutants providing this substrate more frequently, and the charge removal mutant providing it less frequently), owing to their relative abilities to wrap DNA. In the presence of negatively-supercoiled DNA, each gyrase variant exhibits a slight (∼20–40%) decrease in *k*_cat_ with respect to nicked DNA. Although the molecular basis of this decrease is unclear, it may arise from a torsional ‘backpressure’ in supercoiled DNA that makes DNA wrapping (and consequently T-segment capture) less frequent.

Ionic strength is also seen to impact ATPase activity. For wild type gyrase and the charge addition mutants, increasing potassium glutamate concentrations results in a ∼3-fold decrease in *K*_m_ and a modest increase in *k*_cat_ in the presence of either nicked or negatively-supercoiled DNA. The effect of salt concentration on ATPase activity can be summarized by comparing catalytic efficiencies (Supplementary Figure S7C), which shows that there is an ∼3–5-fold increase in *k*_cat_/*K*_m_ when shifting from 50 to 300 mM potassium glutamate. Potassium has been shown structurally to be an important cofactor in ATP binding by the ATPase subunit of gyrase (54). This property could account for the ∼4-fold decrease in *K*_M_ that is observed when the potassium glutamate concentration is shifted from 50 to 300 mM in the presence of DNA, as lower K^+^ levels might be insufficient to saturate the ion-binding site, impairing ATP binding and/or turnover. The R766A mutant breaks this trend, but only because its ATPase rate is comparable to that of the DNA-free enzyme. There is a rough correlation of *k*_cat_/*K*_m_ with the charge addition mutants showing that, in terms of ATPase activity, strengthening wrapping results in higher ATPase efficiency, consistent with the trend observed for *k*_cat_.

### DNA wrapping proficiency anticorrelates with gyrase activities dependent on T-segment engagement *intrans*

**Figure 7.**
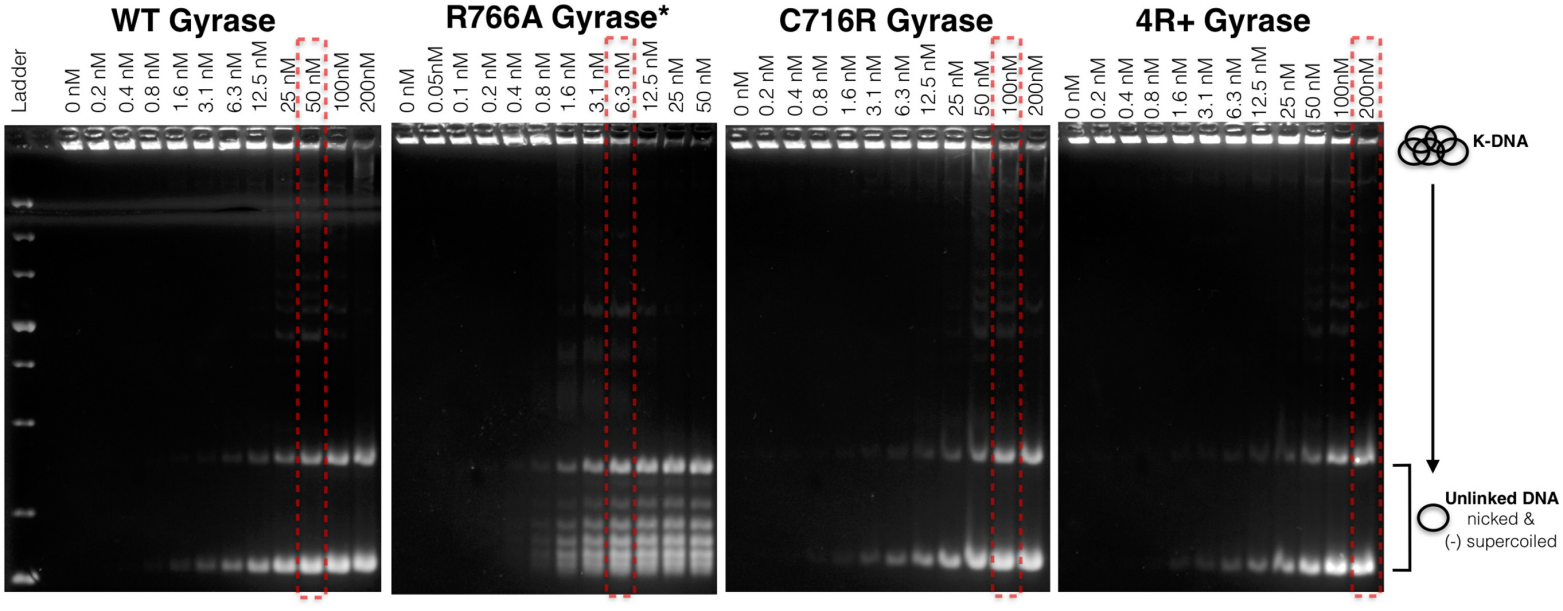
Weakening DNA wrapping by the GyrA CTD enhances the decatenation activity of gyrase. The decatenation of kDNA by *E. coli* gyrase and gyrase mutants using an enzyme concentration titration series is shown. The red dashed boxes highlight enzyme concentrations that produce comparable levels of unlinked kDNA circles. Note that the R766A mutant is capable of only partially supercoiling the unlinked product compared to wild type gyrase and the two charge-addition mutants.

The two major families of bacterial type II topoisomerases, gyrase and topo IV, are distinguished by a respective preference for supercoiling or decatenating DNA (15,55,56). The different properties of these enzymes have been proposed to depend on whether the CTD appended to GyrA (or ParC, the homologous subunit of topo IV) can both bind and wrap or just bind DNA (13–16). The mutant enzymes under investigation here afforded us with an opportunity to test whether DNA wrapping propensity can directly influence the preferential ability of gyrase to decatenate, rather than supercoil, DNA substrates. To probe this question, decatenation assays on kinetoplast DNA (kDNA) were conducted with wild type gyrase and the panel of three CTD mutants. In accord with prior findings (57), *E. coli* gyrase is a relatively weak decatenase, requiring ∼50 nM enzyme to decatenate 50% of 6 nM kDNA substrate in 30 min (Figure 7). As expected, the two charge addition mutants are both less effective at catalyzing DNA decatenation, with the degree of the defect proving more severe for the quadruple 4R+ construct than for the single C716R mutant (roughly 4-fold and 2-fold lower, respectively). By contrast, the charge removal mutant, R766A, is substantially more robust (∼10-fold) than wild type gyrase at decatenating DNA. Although the decatenation activity of the R766A mutant is still significantly less efficient than topo IV, this result accords well with the prediction that DNA wrapping *in cis* disfavors the engagement of a second DNA segment *in trans*, to promote DNA supercoiling over DNA unlinking (4,20,44).

**Figure 8.**
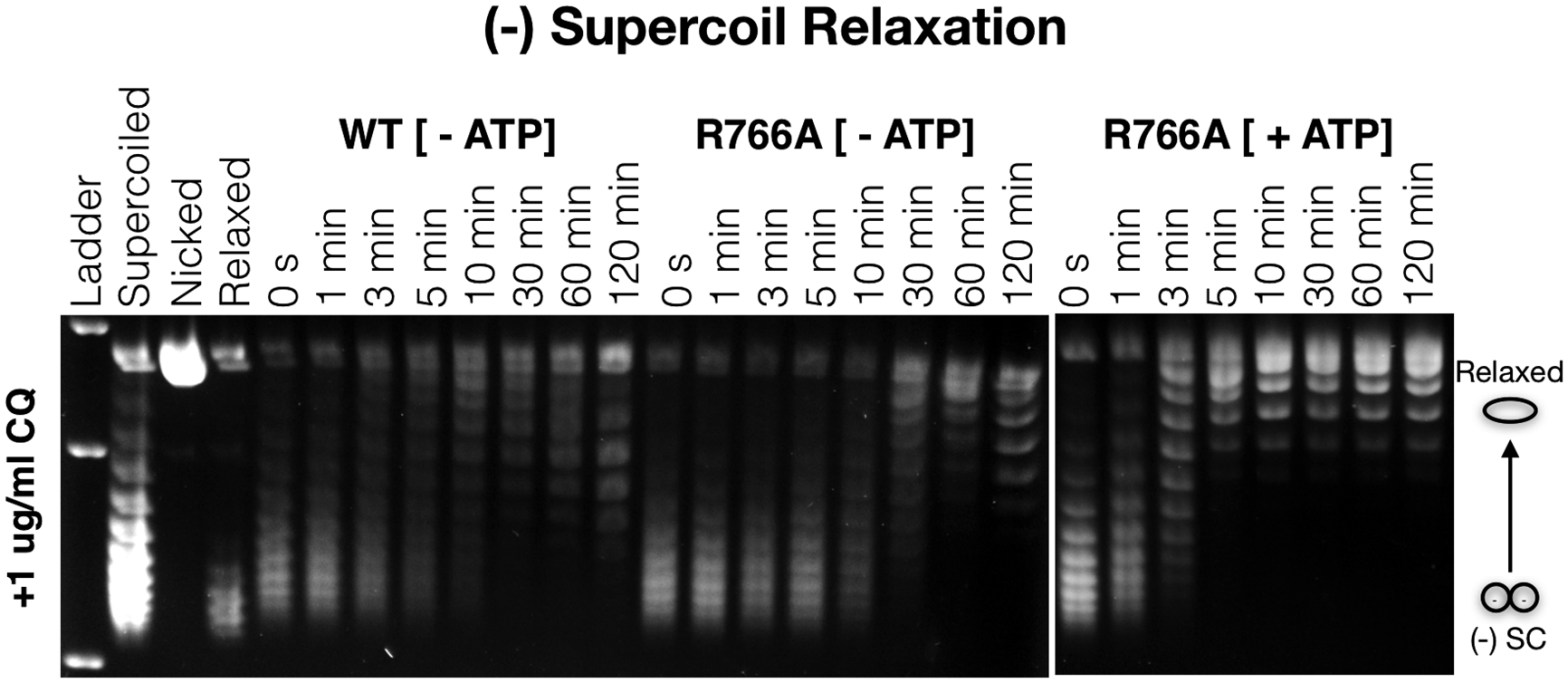
The gyrase R766A mutant exhibits robust negative supercoil-relaxation activity. The ATP-independent and -dependent relaxation of negative supercoils by the R766A gyrase mutant is compared. The molar ratio of enzyme to DNA in all reactions is 1:1.

Given that the R766A mutant seems to more closely approximate topo IV in its ability to decatenate DNA, we were curious whether it might also exhibit an ability to relax negative supercoils. Gyrase can relax negatively-supercoiled DNA in the absence of ATP, although this process is slow and inefficient (58,59). By comparison, *E. coli* topo IV can relax negatively-supercoiled DNA in an ATP-dependent fashion far more rapidly (>1000-fold) (60). To determine whether the R766A mutant can also relax negative supercoils in an ATP-dependent fashion, we performed a timecourse experiment in which enzyme was added at a 1:1 stoichiometric ratio with negatively-supercoiled plasmid with and without ATP. Topoisomers then were resolved on agarose gels containing low amounts of chloroquine (Figure 8). The data show that, like wild type gyrase, the R766A mutant can slowly relax negative supercoils in an ATP-independent fashion. By comparison, the R766A mutant readily relaxes pre-supercoiled DNA in the presence of ATP to a slightly-supercoiled level approaching its setpoint in a time interval of <5 min. Thus, removal of just a single, positively charged amino acid in the GyrA CTD can convert gyrase from a robust supercoiling enzyme that strongly disfavors both DNA decatenation and negative supercoil relaxation to an enzyme that is flipped in its specificities.

## DISCUSSION

The appropriate control of chromosomal supercoiling is critical to cell viability. In bacteria and some archaea, a type IIA topoisomerase known as gyrase negatively-supercoils DNA using an ATP-dependent strand passage mechanism to promote chromosome compaction and aid both transcription and replication (1,3,6). Gyrase orthologs from different prokaryotic species can vary widely in their respective rates and extents of DNA supercoiling (43,48).

It has been hypothesized that the efficiency by which an auxiliary C-terminal domain (CTD) appended to the GyrA subunit of gyrase binds and wraps DNA around itself is a controlling factor in the efficient generation of negative supercoils (4,21,23,30), and that changes in DNA wrapping propensity may serve as an evolutionarily-tunable function that regulates gyrase output. Prior work has shown that mutation of basic amino acids in blade 1 and blade 5 of the *E. coli* and Mtb GyrA CTDs, repspectively, can disrupt gyrase function (42). Here, we show that the removal of a conserved basic residue in blade 5 of the *E. coli* GyrA CTD, along with the addition of charged residues to conserved positions in blades 4 and 6, can systematically and specifically modulate DNA wrapping by gyrase (Figure 2C). A recent structure of the *E. coli* gyrase holoenzyme bound to DNA has revealed that GyrA engages DNA using blades 1, 4, 5 and 6 of the CTD (Supplementary Figure S8) (22). Although individual contacts between residues within the CTD and DNA are not resolved in the structure, the model does help explain why the GyrA Box present in blade 1 is necessary for DNA supercoiling activity and additionally rationalizes the effect of our mutants on DNA wrapping. For example, the C716R mutant likely factiliates wrapping because the engagement of its element (blade 4) with DNA represents the first step in DNA wrap formation. Likewise, the position of the P762R substitution (blade 5), along with the S813R and I823R mutations (blade 6), appear poised to allow additional contacts with the DNA along the CTD surface. In the case of the charge removal mutatants, removing R766 (blade 5) likely destabilizes DNA engagement by the CTD altogether. Blade 2 does not appear to engage DNA in the cryo-EM structure, which may explain why the R612A mutant has no effect on supercoiling activity (Supplementary Figure S1). By comparison, the loss of a CTD-DNA contact in blade 6 (via the R812A mutant) may be masked by the stablilizing effects of the flanking blades with DNA (Supplementary Figure S1). Despite these consistencies, it is unclear why blades 2 and 3—which do not appear to make major contacts with the DNA in the structure—nevertheless possess highly conserved basic residues (R612, R615 and R667) at points where one would expect DNA to bind if wrapping about the CTD were continuous. These sequence relationships indicate that there may exist still other CTD/DNA interactions than those observed thus far.

To more explicitly test how DNA wrapping influences gyrase activity, we examined our mutant gyrase enzymes using DNA supercoiling, decatenation, and ATPase assays. The biochemical data reveal that key gyrase functions indeed correlate with DNA wrapping propensity. For example, a charge removal mutant (R766A) weakly wraps and supercoils DNA when salt concentration is low (Figure 4C), and is further compromised when the ionic strength is increased. This mutant in turn displays a relatively low catalytic efficiency for ATP turnover at low salt, and its performance degrades further when salt levels are increased (Figure 6B). The behavior of the charge-removal mutant indicates that this construct does not wrap DNA as tightly as the native enzyme, and although it can engage DNA to capture a T-segment *in cis* to stimulate ATPase function and conduct strand passage, it can only do so infrequently at low ionic strength and provided there is little to no energetic ‘push-back’ from the superhelical state of its substrate.

The behavior of the R766A mutant contrasts with that of the two charge-addition mutants tested here, C716R and ‘4R+’ (which contains C716R in addition to the changes P762R, S813R, and I823R). Consistent with our initial prediction that selectively incorporating positively-charged residues at key locations on the CTD might strengthen interactions with substrate DNA, both the C716R and 4R+ mutants show an increased propensity to wrap DNA, as evidenced from topology-footprinting assays (Supplementary Figures S2 and S3). At low salt, the supercoiling activity of these mutants is low, yet the ATPase activity is greater than that seen for wild type gyrase (Figures 4A and 6A). These findings indicate that when DNA wrapping is too tight, supercoiling is decoupled from nucleotide hydrolysis leading to increased futile cycling. Comparing their respective supercoiling and ATPase rates, the 4R+ mutant undergoes ∼60 times more ATPase events per successful strand passage event than wild type gyrase at low ionic strength. Interestingly, strengthening DNA wrapping does not substantially change the supercoiling setpoint of *E. coli* gyrase (Figure 5B), suggesting that for this enzyme, the extent to which gyrase adds negative supercoils into a substrate is not limited by stable DNA wrapping. The ATPase data further suggests wrap formation can still occur even when DNA is substantially underwound (as suggested by the high k_cat_ of ATP hydrolysis on a negatively supercoiled substrate, Figure 6A) and that the efficiency with which the enzyme can use ATP to successfully drive strand passage progressively deteriorates as the superhelical density approaches a critical limit (Figure 5).

Given that nucleotide binding is tightly coordinated with DNA wrapping and release in *E. coli* gyrase (13,32,44), it is perhaps unsurprising that perturbing wrapping equilibrium would so markedly affect the rate and catalytic efficiency of DNA supercoiling. Inspection of the data presented here shows that the weaker and stronger wrapping mutants exhibit distinct patterns of behavior. The action of the weakly-wrapping R766A mutant can be modeled in a simple scheme in which wrap formation and T-segment capture in *cis* is less favorable compared to the native enzyme, thus degrading the ability of this construct to introduce negative supercoils (the k_cat_ for ATP-hydrolysis by the mutant is correspondingly similar to a DNA-free state because T-segment capture is also necessary for efficient ATPase dimerization and hydrolysis (7,29,30,53)). Nonetheless, the inability of the R766A CTD to stably wrap DNA does favor T-segment selection *in trans*, thereby improving this mutant's ability to decatenate DNA and relax negative supercoils in an ATP-dependent fashion (Figures 7 and 8). Thus, on relaxed or moderately-underwound substrates, the R766A construct operates as a conventional gyrase, whereas on more negatively-supercoiled substrates it functions as an ATP-dependent relaxase. It is noteworthy that a single point change to the GyrA CTD is sufficient to generate an enzyme with hybrid gyrase/topo IV-like properties reflective of evolutionarily-distant homologs, such as *M. tuberculosis* gyrase. This behavior accords with the kinetic competition model proposed by Nöllmann *et al.*, whereby gyrase output is defined by partitioning between three modes of action: (i) ATP-dependent strand passage of a proximal (*cis*) T-segment (due to wrapping by the CTD), (ii) ATP-dependent strand passage of a distal (*trans*) T-segment (no wrap, but perhaps CTD•DNA engagement), and (iii) ATP-independent strand passage of negative superhelical crossings (34).

Considering the R766A data alone and the kinetic competition model proposed by Nöllmann *et al.*, one would expect that the tighter wrapping mutants would have more robust supercoiling activities and a higher setpoint than wild type gyrase. As our data shows, this is not the case. The most likely reason for the disparity is that gyrase becomes less efficient and more prone to futile cycling as its DNA substrate becomes progressively more negatively supercoiled (29,30,35,44,50). This behavior is evident in Figures 5A and 6, which show a slowing of supercoiling rate on negatively-supercoiled substrates (comparing the ΔLk between time points 1–5 minutes and 10–30 min) despite retaining high ATPase activity. To explain this phenomenon, it has been suggested that gyrase operates as a nucleotide-dependent trap, and that the decrease in energetic coupling observed on more negatively-supercoiled substrates is due to a lowered frequency by which GyrB engages a T-segment (30,34,61). This however appears to not be the case. Although our observed correlation between the ATPase rate and a propensity to form a DNA wrap on a relaxed substrate comports with such a model (Figures 3 and 6), the corresponding decrease seen for the rate of ATP-dependent strand passage does not (Figure 4). If the ATP-dependent trap model were wholly correct, then the C716R and 4R+ mutants should be more efficient at negatively-supercoiling DNA (under relaxed conditions) and less prone to slippage (under supercoiled conditions) than wild type gyrase, not the opposite.

If the propensity to form a DNA wrap does not explain the observed changes in ATPase-strand passage coupling efficiency, then what is the basis for this behavior? The observations reported here are most consistent with the model of Kampranis *et al.*, in which T-segment capture occurs readily, even on highly negatively-supercoiled DNA, but where the likelihood of completing directional strand passage is largely dependent on the energetic cost of adding two additional supercoils to the substrate for *E. coli* gyrase (35,40). As a DNA substrate becomes increasingly supercoiled, the energetic demand to undergo an additional round of strand passage scales quadratically in a manner similar to a Hook's law spring (40), so that strand passage and cycle completion become increasingly less likely as opposing superhelical tension builds, giving rise to futile cycling. The resultant picture is one of a climbing energy landscape in which cycle progression is hindered by both the energetics of wrap formation *and* the probability of transitioning into the final product state following the addition of two negative supercoils. The lowered enzymatic efficiency of the charge addition mutants examined here is consistent with such a model: as both the C716R and 4R+ mutants stabilize the pre-passage wrapped state, they add to the energetic barrier for completing the strand passage reaction, shunting the enzyme toward futile cycling and resulting in slower supercoil generation. In classical enzymology terms (E+S → ES → ES* → E+P), this is the equivalent of the tighter-wrapping mutants stabilizing the enzyme-substrate complex, while the energetics of the transition state remains the same (or increases), resulting in a higher overall barrier to the reaction. Consistent with this idea, specific DNA sequences known as strong gyrase sites—which promote the formation of a stably-wrapped complex—have been shown to decrease *in vitro* supercoiling rates relative to non-specific sequences (62,63).

The effect of potassium concentration (as well as other solutes, e.g., see (51)) on both the supercoiling efficiency (Figure 4) and setpoint (Figure 5) of gyrase is also consistent with the idea that the enzyme is sensitive to the free-energy state of its supercoiled product. Cations have long been known to increase DNA flexibility and lower the free-energy state of supercoiled DNA topoisomers (40,51). In terms of gyrase's supercoiling reaction, increasing potassium glutamate concentration reduces the energy barrier to a given strand passage event. This effect may account for why a substantial increase in supercoiling setpoint is seen upon going from 80mM to 300mM KGlu, not only for wild type gyrase and our charge-addition mutants (Figure 4), but for *M. tuberculosis* gyrase as well (Supplementary Figure S6). Moreover, a decrease in the effective stiffness can account for the observed increase in supercoiling setpoint at high salt (Figure 5B): if DNA supercoiling obeys Hooke's Law, increasing the potassium concentration reduces the effective Hook's law spring constant *k* (40) that approximates the energetics of supercoiling. Thus, the increase in potential energy (*U* = 1/2*kx*^2^) upon each additional twist is less steep in the presence of high potassium, resulting in the ability of gyrase to incorporate more DNA supercoils compared to low ionic strength. Experiments have been conducted under a variety of ionic conditions to test how the presence of Na^+^, Mg^2+^, and spermidine^3+^, may affect the energetics of supercoiling (64). Interestingly, the effective spring constant was found to be ∼50% greater under low salt conditions versus conditions containing high concentrations of spermidine or magnesium (40,64), consistent with the ∼50% increase in setpoint at high salt (Figure 5B).

The inability of the charge addition mutants to supercoil DNA to a greater extent than wild type gyrase may be explained from thermodynamic considerations, if the native enzyme is already operating at a thermodynamic limit. The strength of DNA wrapping by the CTD does not affect the underlying thermodynamics of the starting DNA substrate and its supercoiled product, but does affect the intermediate states formed during the supercoiling reaction and hence the enzyme kinetics as well. From this perspective, the supercoiling setpoint of a given gyrase can be limited by the amount of energy that it can harness from the ATP-hydrolysis reaction (a notion espoused previously by others; e.g., see Cullis *et al.* and Bates *et al.* (36,65)), but only provided that the stability of DNA-wrapping exceeds a certain energetic threshold that can out-compete off-pathway processes such as the ATP-dependent relaxation of negative DNA supercoils. The extent to which DNA wrapping strength might also modulate the relaxation of positive DNA supercoils remains to be determined (66).

**Figure 9.**
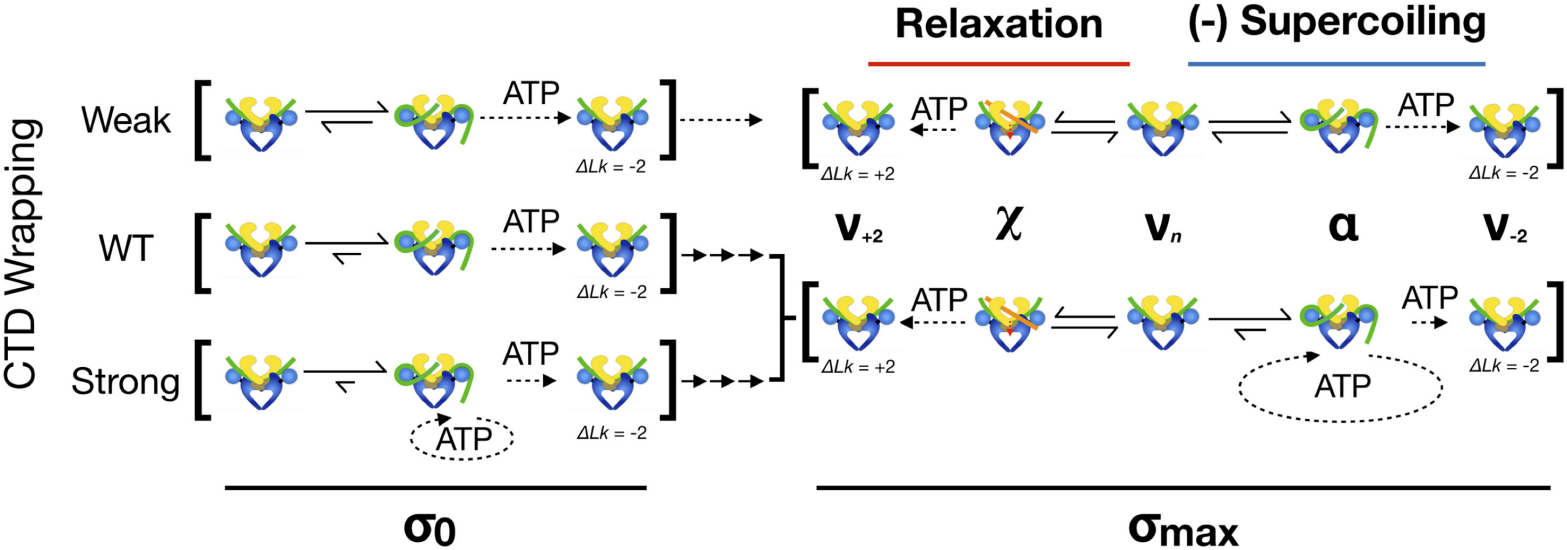
Simplified kinetic scheme for DNA supercoiling. DNA gyrase follows two distinct ATP-dependent pathways—(1) negative supercoiling through T-segment engagement in *cis* (***α***-state) and (2) supercoil relaxation through T-segment engagement in *trans* (***χ***-state). The likelihood of transitioning between each state is dependent on both the ability to form a chiral wrap/engage a crossover and the superhelical density (***σ****)* of the DNA substrate. **σ_0_**: On relaxed substrates, the relaxation pathway is negligible given that the energy barriers to both DNA wrapping and strand passage are low and that there is a low likelihood of engaging a crossover in *trans*. Wrapping contributes stability to the ***α***-state, and impedes strand passage only when wrapping is overly strong, in which case futile cycling occurs. **σ_max_**: At its set-point, the relaxation and negative-supercoiling modes of gyrase are in competition regardless of wrapping proficiency. As negative supercoils accumulate, the stability of the ***α***-state decreases while the likelihood of ***χ***-state formation increases. Similarly, the efficiency of strand passage is correlated with the free-energy difference between the starting σ value and the product of the reaction. Thus, as supercoils accumulate, the probability of adding two negative supercoils decreases rapidly whereas that of the relaxation reaction increases. For a weakly-wrapping gyrase, ***α***-state formation is disfavored and becomes increasingly difficult as supercoiling progresses until it competes equally with ***χ***-formation. In the case of native *E. coli* gyrase or a more tightly-wrapping construct, ***α***-state formation may remain favorable at the setpoint, but the free energy required for completing the overall cycle and adding two negative supercoils is very high (on the order of ∼10 s of *kT*), and strand passage following ***α***-formation is inefficient, manifesting as futile cycling.

In summary, the biochemical data obtained for the weaker and tighter DNA-wrapping CTD mutants suggest that the supercoiling rate and extent of gyrase is bounded by two different regimes, one where DNA wrapping strength is weak and therefore limiting in terms of supercoiling rate and setpoint, and another where DNA wrapping is strong and the coupling efficiency between ATP turnover and strand passage governs the two properties (Figure 9). Both supercoiling rate and setpoint are likely to be important to differing extents for a given species of gyrase. Such a duality would help explain why the 4R+ mutant, for example, appears to incorporate one or two additional supercoils relative to wild type and C716R (Figure 5B) – at or around its setpoint, wild type gyrase is likely less able to wrap DNA as readily as at lower superhelicity levels, thereby increasing the likelihood of capturing a T-segment in *trans* to promote supercoil relaxation (34). It is conceivable that the high setpoint of *E. coli* gyrase may be the product of stable wrap formation at high superhelical densities, sterically precluding DNA relaxation (although at the expense of futile cycling and ATP-consumption), thus unifying both the Nöllmann and Kampranis models. Along these lines, the marginally higher setpoint of the 4R+ mutant is consistent with the idea that *E. coli* gyrase operates at or near a supercoiling optimum governed by the efficiency of the ATP-driven strand passage reaction (35,36,50,65). Collectively, these studies stand as a complement to prior efforts showing that evolutionary differences in the C-terminal tail of GyrA (43,67) and GyrB (67,68) can affect the rate and extent of DNA supercoiling by gyrase. Future work will be needed to further refine the extent to which different aspects of the complex gyrase holoenzyme have evolved to work in opposition and conjunction with one another to appropriately sense and modulate supercoiling homeostasis across disparate prokaryotic species.

## Supplementary Material

gkz1230_Supplemental_File
