# Supplementary material for: Modulated control of DNA supercoiling balance by the DNA-wrapping domain of bacterial gyrase
Source: Nucleic Acids Res. 2020 Jan 17;48(4):2035–49. doi: 10.1093/nar/gkz1230 (PMC7038939; doi:10.1093/nar/gkz1230)
Supplement: gkz1230_Supplemental_File [file gkz1230_supplemental_file.pdf]

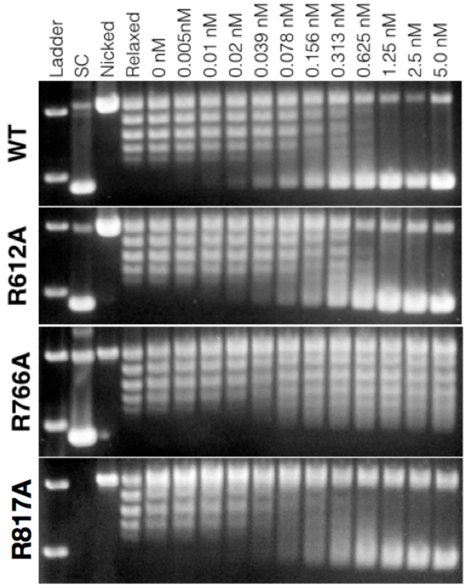

Supplemental Figure 1: Preliminary screening of gyrase charge removal mutants in GyrA

Arginine residues within the GyrA box motifs of blades 2, 5, and 6 (**Figure 2B**) were mutated to alanine and tested for their effect on DNA supercoiling (**Materials and Methods**). R766A was dramatically less effective at DNA supercoiling than wildtype gyrase, undergoing only a few rounds of supercoiling before stalling. R612A and R812A were comparable to wild type and not examined further.

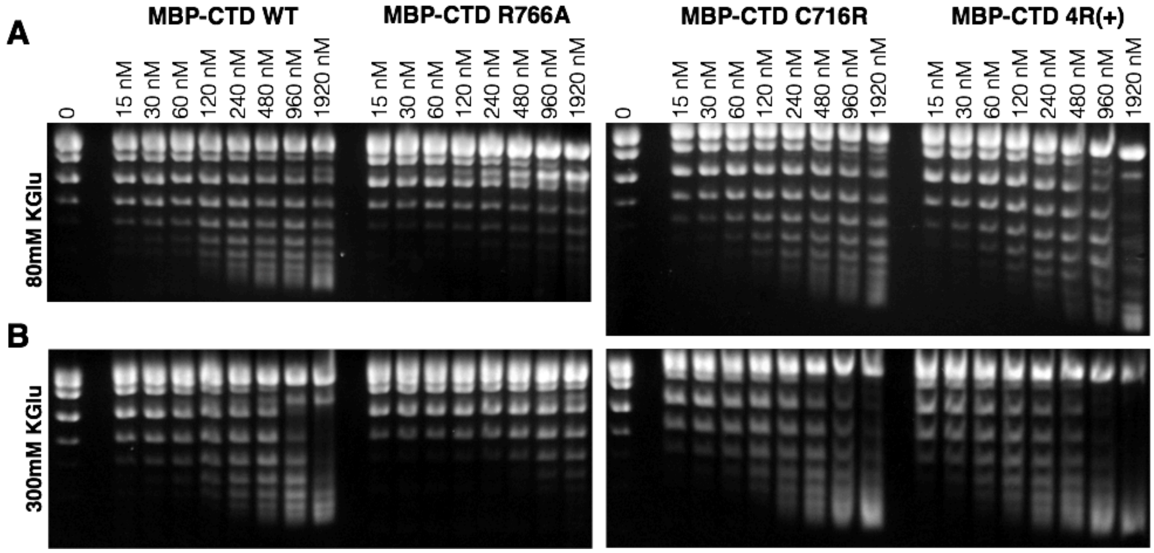

Figure S2: Topology footprinting of GyrA CTD variants

A) DNA wrapping by the CTD alone in the presence of 80mM potassium glutamate. The CTD was analyzed as an MBP-fusion to prevent aggregation that was seen for the isolated domain (**Methods**).

B) Repeat of the DNA wrapping assay in the presence of 300mM potassium glutamate.

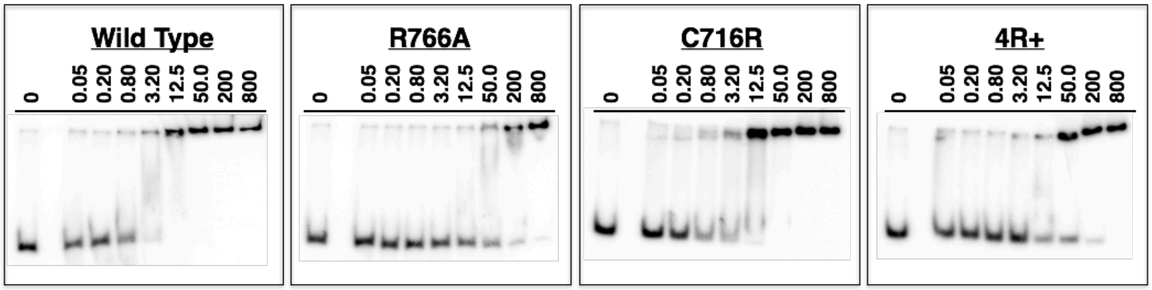

Figure S3: EMSAs of DNA gyrase and gyrase mutants

Gyrase concentrations are in nanomolar units. DNA binding by the C716R and 4R+ mutants are comparable to wild type (within a factor of 4) while the R766A mutant appears to bind DNA about 20-fold more weakly than the native enzyme. The approximate dissociation constants for all of the gyrase variants (as estimated by 50% binding of substrate) are below the binding site concentrations used in wrapping/supercoiling/ATPase assays, indicating that functional defects observed for the mutants are not due to an inability to associate with plasmid substrate.

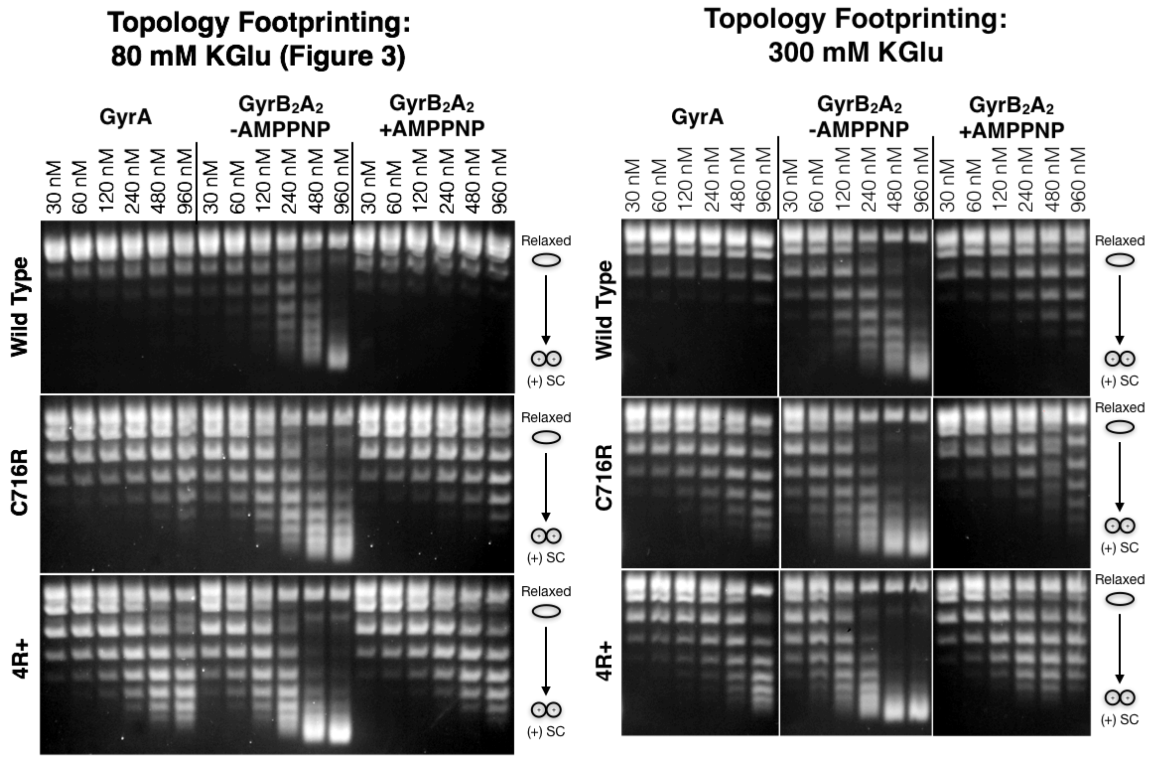

Figure S4: DNA wrapping is preserved at 300mM KGlu

Topology footprinting assays comparing the DNA wrapping propensities of mutant and wildtype GyrA subunits and gyrase holoeznymes under different KGlu concentrations. The data from Figure 3 (80 mM KGlu) are shown side-by-side with the elevated salt experiments (300 mM KGlu).

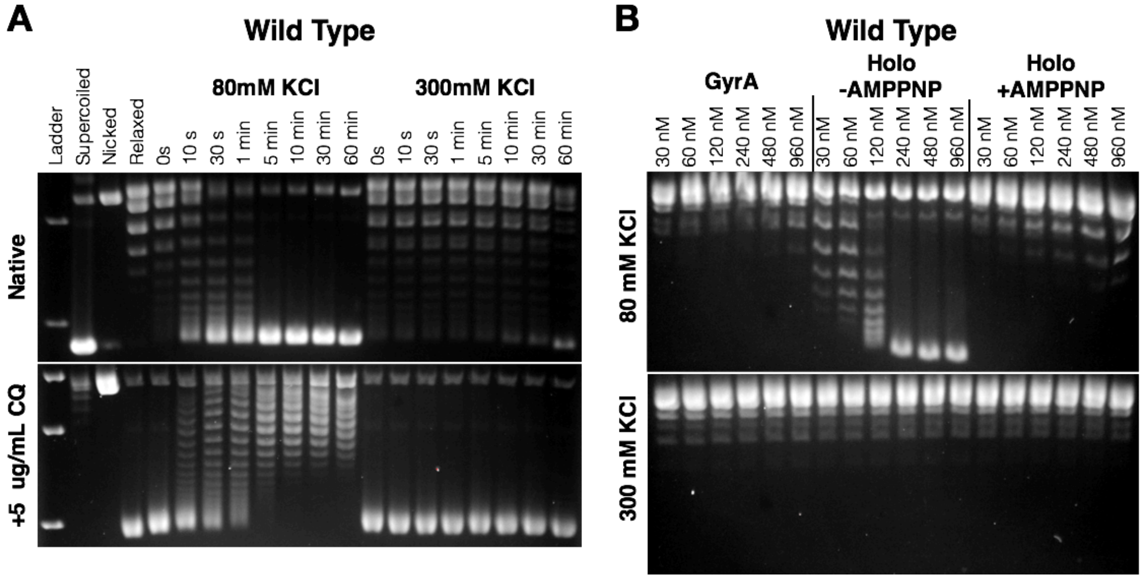

Figure S5: High potassium chloride concentrations impair DNA supercoiling and wrapping by *E. coli* gyrase

A) Supercoiling timecourses in the presence of KCl reveal high activity in 80mM salt but significant impairment at 300mM.

B) Topology footprinting by gyrase in the presence of potassium chloride reveals a complete loss of DNA wrapping at 300mM KCl. The molar ratio of enzyme to DNA in all reactions is 1:1.

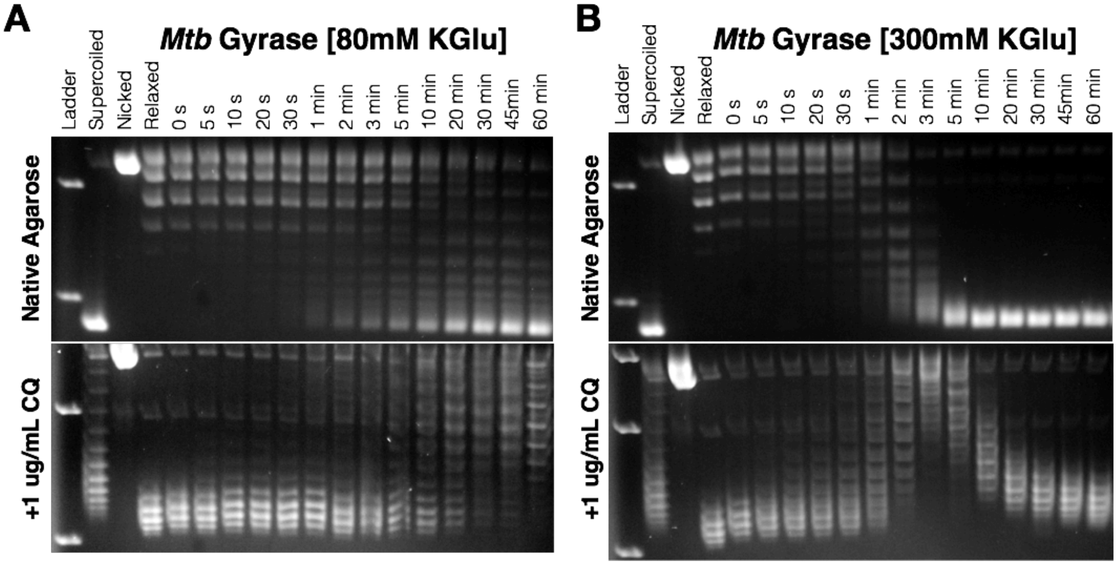

Figure S6: High levels of KGlu enhance DNA supercoiling by *Mtb* gyrase

A) DNA supercoiling timecourse for *Mtb* gyrase in 80mM potassium glutamate.

B) DNA supercoiling timecourse for *Mtb* gyrase in 300mM potassium glutamate. The molar enzyme:DNA ratio in all reactions is 1:1.

		50mM KGlu		300mM KGlu	
		k_{cat} (s^{-1})	K_M (μM)	k_{cat} (s^{-1})	K_M (μM)
Nicked pSG483	**WT**	1.9 ± 0.2	320 ± 90	2.4 ± 0.1	120 ± 10
	**R766A**	0.9 ± 0.1	410 ± 120	0.5 ± 0.1	860 ± 270
	**C716R**	3.1 ± 0.2	420 ± 60	3.5 ± 0.1	140 ± 20
	**4R+**	3.7 ± 0.2	490 ± 80	3.9 ± 0.2	100 ± 20
(-)sc pSG483	**WT**	1.1 ± 0.1	350 ± 70	1.5 ± 0.1	180 ± 20
	**R766A**	0.4 ± 0.1	400 ± 150	0.8 ± 0.1	1300 ± 320
	**C716R**	2.3 ± 0.2	700 ± 130	2.6 ± 0.1	150 ± 20
	**4R+**	2.3 ± 0.1	340 ± 60	2.7 ± 0.1	150 ± 20
No DNA	**WT**	0.7 ± 0.1	980 ± 60	0.6 ± 0.2	4100 ± 1900
	**R766A**	0.7 ± 0.1	830 ± 90	0.4 ± 0.1	980 ± 110
	**C716R**	0.7 ± 0.1	1360 ± 70	0.6 ± 0.1	700 ± 40
	**4R+**	0.9 ± 0.1	1000 ± 200	0.4 ± 0.1	450 ± 80

Supplemental Table 1: ATPase parameters for wildtype gyrase and select CTD variants

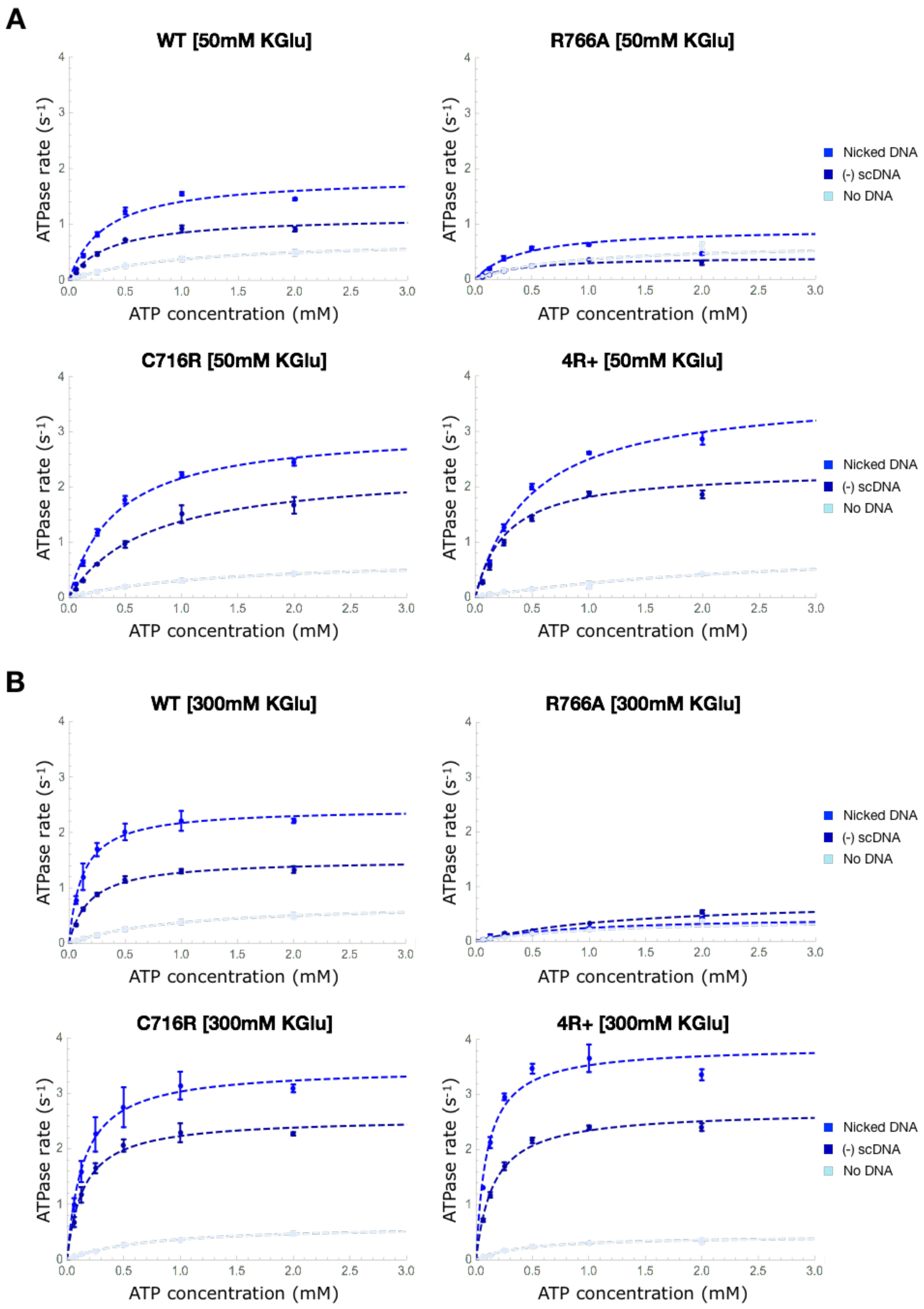
A
WT [50mM KGlu]
R766A [50mM KGlu]
C716R [50mM KGlu]
4R+ [50mM KGlu]
B
WT [300mM KGlu]
R766A [300mM KGlu]
C716R [300mM KGlu]
4R+ [300mM KGlu]
ATPase rate (s-1)
ATP concentration (mM)
Nicked DNA
(-) scDNA
No DNA
0
1
2
3
4
0.0
0.5
1.0
1.5
2.0
2.5
3.0

C

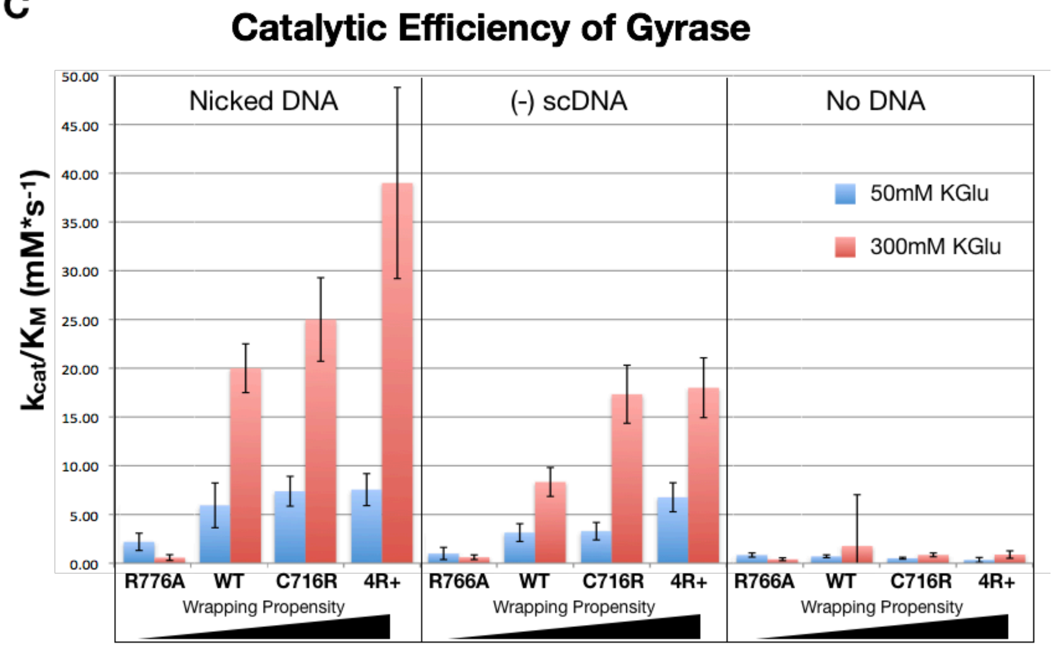

Figure S7: Representative ATPase data for wildtype gyrase and select CTD variants

A) ATPase data and Michaelis-Menten fits for reactions conducted with 50 mM KGlu.

B) ATPase data and Michaelis-Menten fits for reactions conducted with 300 mM KGlu. For both panels, error bars are expressed as the standard deviation (n=3) for the rate (ATP per enzyme per second) for a given ATP concentration. All experiments were conducted in triplicate. The molar enzyme:DNA ratio of "binding sites" (300bp) in all reactions is ~1:5. DNA concentrations are well above the reported K_D of the enzyme (~10 nM).

C) Catlytic efficiency of gyrase mutants as a function of salt and DNA topology. Differences in catalytic efficiency is largely attributable to the salt affect on K_M.

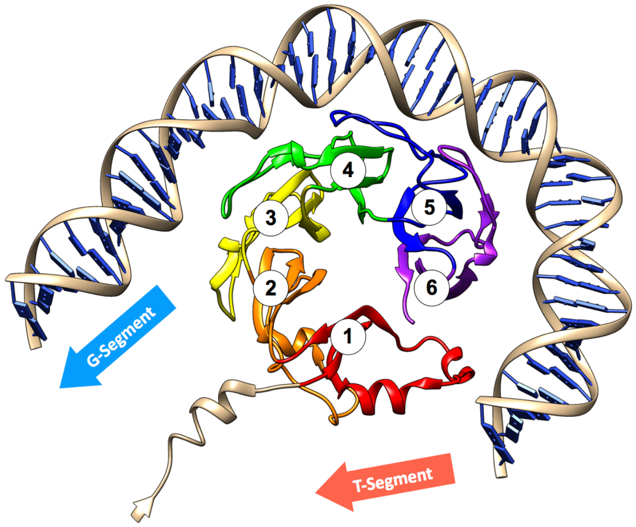

Mutation	R/K Conservation	Result
**Φ → R**		
**C716 (Blade 4)**	89.5%	Stronger Wrapping
**P762 (Blade 5)**	42.2%	(w/ C716R) Stronger Wrapping
**S813 (Blade 6)**	35.7%	
**I823 (Blade 6)**	41.4%	
**R → A**		
**R612 (Blade 2)**	76.5%	None
**R766 (Blade 5)**	98.5%	Weaker Wrapping
**R817 (Blade 6)**	97.8%	None

Figure S8: Individual blade contributions to CTD-DNA wrapping

DNA engagement by the GyrA CTD (PDB ID: 6RKW) proceeds from blade 3 to 6 with the blade 1 culminating the DNA wrap. This CTD orientation may explain why specific CTD-residues contribute more to DNA wrapping than others.

Supplementary Methods:

Electrophoretic Mobility Shift Assays (EMSAs)

A 200bp DNA substrate was radiolabled with ^{32}P using T4 polynucleotide kinase (NEB) and ATP, [γ-^{32}P] (Perkin-Elmer). The reaction was cleaned up using a G-50 desalting spin column (GE Healthcare Life Sciences). A titration series (0 to 800nM) of gyrase holoenzyme was combined with 0.5nM ^{32}P-labeled DNA substrate in a buffer containing 50mM potassium glutamate, 6mM $MgCl_2$, 0.1 mg/mL BSA, 2mM DTT, 50mM Tris-HCl pH 7.9, and 10% glycerol. Reaction volumes of 20uL were incubated at 25°C for 10 min, prior to loading on to an 8% acrylamide (29:1) TBE gel. Gels were run for 2.5 hrs at 60V in TBE buffer. Following the separation of the bound and unbound species, gels were stored with a phosphorimaging plate overnight and imaged using a Typhoon FLA 9500 biomolecular imager.

DNA Sequence:

5'-gggaaacctgtcgtgccagctgcattaatgaatcggccaacgcgcggggagaggcggtttgcgtattgggcgctcttccgcttcctcgctcactg
actcgctgcgctcggtcgttcggctgcggcgagcggtatcagctcactcaaaggcggtaatacggttatccacagaatcaggggataacgcagga
aagaacatgt-3'
